# Occurrence and Distribution of *Vibrio alginolyticus* in Shellfish

**DOI:** 10.3390/foods15101642

**Published:** 2026-05-08

**Authors:** Temitope C. Ekundayo, Frederick T. Tabit

**Affiliations:** Department of Life and Consumer Sciences, University of South Africa, Cnr Christiaan De Wet and Pioneer Ave, FL, Private Bag X6, Roodepoort 1710, South Africa

**Keywords:** molluscs, crustaceans, food safety, clams, mussels, oysters

## Abstract

*V. alginolyticus* (VAlg) is one of the three *Vibrio* species causing human vibriosis. Its presence in shellfish constitutes a potential health risk. Thus, this investigation aimed to determine the prevalence and distribution of VAlg in 18,544 shellfish across geographies using standard protocols and random intercepts/mixed-effects regressions. The global VAlg prevalence in shellfish was 19.70% (95% CI: 13.54–27.75). VAlg pooled prevalence was significantly different (*p* < 0.0001) by shellfish type ($\chi_{20}^{2}$ = 238.48), species ($\chi_{74}^{2}$ = 440.34), genus ($\chi_{51}^{2}$ = 414.37), VAlg detection methods ($\chi_{10}^{2}$ = 150.43), nation ($\chi_{30}^{2}$ = 632.27), and continent ($\chi_{4}^{2}$ = 33.81). Europe and South America showed the highest pooled VAlg contaminated rates in shellfish (27% and 32%, respectively) but Asia had a low rate (~4%). By shellfish type, gastropods (snails) topped the list with 50%, followed by bivalves (29%). Among shellfish species, the VAlg rate declined from 67% to 5% (k ≤ 6), but with a more stable prevalence in *Litopenaeus vannamei* (19.02%, k = 11) and *Crassostrea gigas* (15.18%, k = 9). Edible oysters and clams had lower VAlg pooled rates (4–15%). Targeted culture with MALDI-TOF or species-specific qPCR detected VAlg in 100% of tested shellfish samples. By contrast, conventional phenotypic tests detected less VAlg in shellfish yielding 20% by general biochemical/API tests, 8.8% by generic PCR, and near-zero (0.3–3%) by multiplex PCR or MALDI-TOF/PCR. Both the multiplicative ($\beta_{0}=-3.28\pm 3.76,\;F_{54;125}$ = 3.01, *p* = 0.001) and additive ($\beta_{0}=-1.89\pm 1.47,\;F_{31;148}$ = 3.83, *p* = 0.001) interactions of nation and sample size explained 73.14% and 58.04% of the true variance in VAlg prevalence in shellfish. Other factors include detection techniques ($R^{2}$ = 46.59%, $\beta_{0}=-1.63\pm 0.83,\;F_{10;169}=11.16$, *p* = 0.001), nation ($R^{2}=37.72$ = 1.89, *p* = 0.006), medium $R^{2}$ = 19.42%, $\beta_{0}=2.24\pm 1.85,\;F_{15;164}$ = 1.69, *p* = 0.03), shellfish type ($R^{2}$ = 16.99%, $\beta_{0}=-3.90\pm 1.32,\;F_{20;159}$ = 1.34, *p* = 0.12), and continent ($R^{2}$ = 15.29%, $\beta_{0}=-1.81\pm 0.41,\;F_{4;175}$ = 5.90, *p* = 0.001). In conclusion, the study reveals substantial occurrence of VAlg in shellfish worldwide, with notable regional and species-specific hotspots. Harmonized and molecular-based surveillance of VAlg in shellfish linked to food safety criteria will be essential to manage its emerging threat.

## 1. Introduction

*Vibrio alginolyticus* is a halophilic, Gram-negative bacterium ubiquitous to brackish and marine waters, where it colonizes fish, shellfish, and other aquatic animals [1,2]. *Vibrio alginolyticus* is the most frequently isolated “other” *Vibrio* species aside from *V. parahaemolyticus* in seafood both in-harvest and post-harvest shellfish samples in North America, Europe, and Asia [2]. For example, seasonal monitoring of *Vibrio* in mud and seawater in South Korea found *V. alginolyticus* year-round (6262/10,983 *Vibrio* isolates) when *V. vulnificus* and *V. cholerae* only appeared in summer [3]. Similarly, surveys of bacterial contaminants in US and European shellfish and farmed Southeast Asian shrimps report *V. alginolyticus* at harvest with detection rates ranging from 5 to 50% [2,3]. Generally, *V. alginolyticus* thrives in warm waters globally and filter- and detritus-feeding shellfish readily bioaccumulate *V. alginolyticus* from the aquatic environments, reflecting its broad environmental distribution [2,3].

Recent studies have confirmed the wide geographic presence of *V. alginolyticus* in shellfish and shellfish beds [4,5,6,7,8,9,10,11,12,13,14,15,16]. In addition, *V. alginolyticus* has emerged as a pathogen in diverse aquatic species in many regions and it has caused mass mortalities and infections in Pacific oysters in China, and Mediterranean and Asian finfish (Turkey, India, Taiwan), respectively [12,14,15,16]. *V. alginolyticus* is also frequently ranked behind only *V. parahaemolyticus* among *Vibrio* isolates in surveys of bacterial contaminants in retailed seafoods [17]. Zeidler et al. detected *V. alginolyticus* in 42% of *Vibrio*-positive shrimp and mussel samples from German markets [17]. Many studies have shown *V. alginolyticus* presence across all continents in wild and farmed shellfish, with its prevalence influenced by local temperature, salinity, and season [12,13,14,15,16,18]. The data from these previous studies therefore indicated that *V. alginolyticus* is a common member of the *Vibrio* community in marine and aquatic food species worldwide [12,13,14,15,16].

Despite the ubiquity of *V. alginolyticus* in brackish/marine environments and aquatic animals, *V. alginolyticus* has been understudied and under-reported in shellfish across many nations and regions. Yet, *V. alginolyticus* is well-known for causing opportunistic infections (otitis, dermatitis, and wound infections) in humans, but its role in seafood- and shellfish-borne illness remains poorly characterized [19,20,21]. The CDC lists *V. alginolyticus* among the top three *Vibrio* species causing human infections in the USA [7,22,23]; nevertheless, its actual food-borne cases are rarely confirmed. For instance, Zeidler et al. note that “conclusive studies on food-borne infections caused by *V. alginolyticus* are scarce” [17]. In truth, genetic tools, and surveillance studies of bacterial contaminants in seafood- or shellfish-borne infections have traditionally focused on *V. parahaemolyticus* and *V. vulnificus*, so the true incidence of *V. alginolyticus* gastroenteritis or vibriosis may be underestimated. Emerging challenges of widespread antibiotic-resistant genes and climate change-driven warming are expected to amplify the concern for expanding *V. alginolyticus* host ranges in shellfish and its seasonal distribution [1]. For this reason, Sun et al. emphasized a widespread occurrence of *V. alginolyticus* in seafood and advocated its vigilant monitoring to strengthen food safety [1]. In summary, *V. alginolyticus* is a global prevalent pathogen in shellfish, poses documented health risks (especially for vulnerable consumers), and yet significant gaps remain in our understanding of its epidemiology, pathogenic potential, and distribution in shellfish [2,17,22,23]. Therefore, this investigation aimed to determine the prevalence and distribution of *V. alginolyticus* in shellfish across geographies and identify the numerous factors moderating its prevalence in shellfish.

## 2. Materials and Methods

### 2.1. Data Strategy and Inclusion Criteria

*V. alginolyticus* contamination original research data in shellfish were sought in Scopus, PubMed, and Web of Science. We applied a topic (title–abstract–keywords) search using “alginolyticus AND (abalone* OR bivalve* OR crab* OR crawfish* OR crayfish* OR clams OR cockle* OR conch* OR cuttlefish OR gooseneck* OR lobster* OR limpet* OR mussel* OR mollusc* OR oyster* OR octopus* OR periwinkle* OR prawn* OR shrimp* OR krill* OR barnacle* OR shellfish* OR seafood* OR squid* OR scallop* OR whelk*)”. *Vibrio alginolyticus* was specified as “alginolyticus” in the search for specificity without undermining recovery. The search targeted published peer-reviewed articles spanning the inception of the databases to 26 August 2025 regardless of the language. The Supplementary Materials contained database-specific queries. *V. alginolyticus* contamination of shellfish is defined as its specific detection via standard culture/culture-independent assays. The manuscript is an intrinsic part of the project approved by the College of Agriculture and Environmental Sciences_Health REC, University of South Africa, with the ethical clearance reference number 2025/CAES_HREC/6998. Notwithstanding this, no new animal data was collected in this investigation.

The study inclusion criteria included access to the final selected studies’ full texts, *V. alginolyticus* incidence data, the studies’ primary descriptors (author/year/nation), methodological details (growth medium/detection assay), but details about shellfish species/type were regarded as optional data. All secondary/gray literature sources were excluded.

### 2.2. Data Processing Details

We merged the search data outcomes in Zotero (version 7.0.13) which involved duplicates being removed from the collection. The merged collection was then retrieved for title–abstract screening in Excel 2016. The screening targeted original research articles for inclusion based on PRISMA 2021 guidelines. The full texts of the eligible studies following this stage were downloaded, read, and research data reported in them were mined independently by two reviewers (one author and an external consultant) into two data forms. We translated non-English records into English by using Google Translate. Plot data were also mined by using Plotdigitizer Version 3.1.6 agency. The reviewers validated final data; where disagreement occurred, it was resolved through consensual discussion. The process is schematically represented in Figure S1.

The primary data elements to achieve the aim of the study included citation (author/year), *V. alginolyticus* incidence data, shellfish type/species, culture medium, detection method, and nation. Optional/derivable data elements were shellfish class/genus, and continent (using Seven-Continent Scheme). Also, studies missing species data were designated “species not reported” for the species-specific prevalence calculations to ensure statistical accuracy.

### 2.3. Statistical Analysis and Synthesis

*V. alginolyticus* data from 18,544 shellfish mined from seventy-five studies (Table 1) were first logit-transformed [24], fitted to study-level random intercepts regression based on maximum-likelihood link function [25] and to the Egger’s regression [26]. For sensitivity analyses, we fitted the data firstly by removing all outlying studies [27], and secondly, to leave-one-out analytic model [25]. In the second part, we fitted logit-transformed 180-shellfish-type/species-level (disaggregated dataset) mixed-effects regressions to assess *V. alginolyticus* rates by shellfish type/species, detection assay, shellfish class/genus, medium, and geography (nation/continent) [28]. In both cases, the associated heterogeneity was evaluated using maximum-likelihood estimator [29]. The disaggregated dataset entries were derived from the primary included studies such that a study that assayed multiple shellfish types/species contributed more than one shellfish type/species independent dataset to the disaggregated data (Table S1). In all models, zero-event studies were mechanically managed by using 0.5 continuity adjustment across all data points. Further, we identified and quantified the roles of shellfish species, sample size, medium, name, detection assay, class/genus, and geography (nation/continent) to *V. alginolyticus* occurrence variance in shellfish by fitting the shellfish-type/species-level dataset to 1000-permutation-based univariate/bivariate mixed-effects meta-regressions using maximum-likelihood link function with continuous data coded as numerical variables and categorical data coded as discrete variables as the case may be [30,31]. The models were fitted in R v. 4.5.1 using metafor 4.8-0/meta 8.2-0 packages.

## 3. Results

### 3.1. V. alginolyticus Global Prevalence in Shellfish

**Figure 1:**
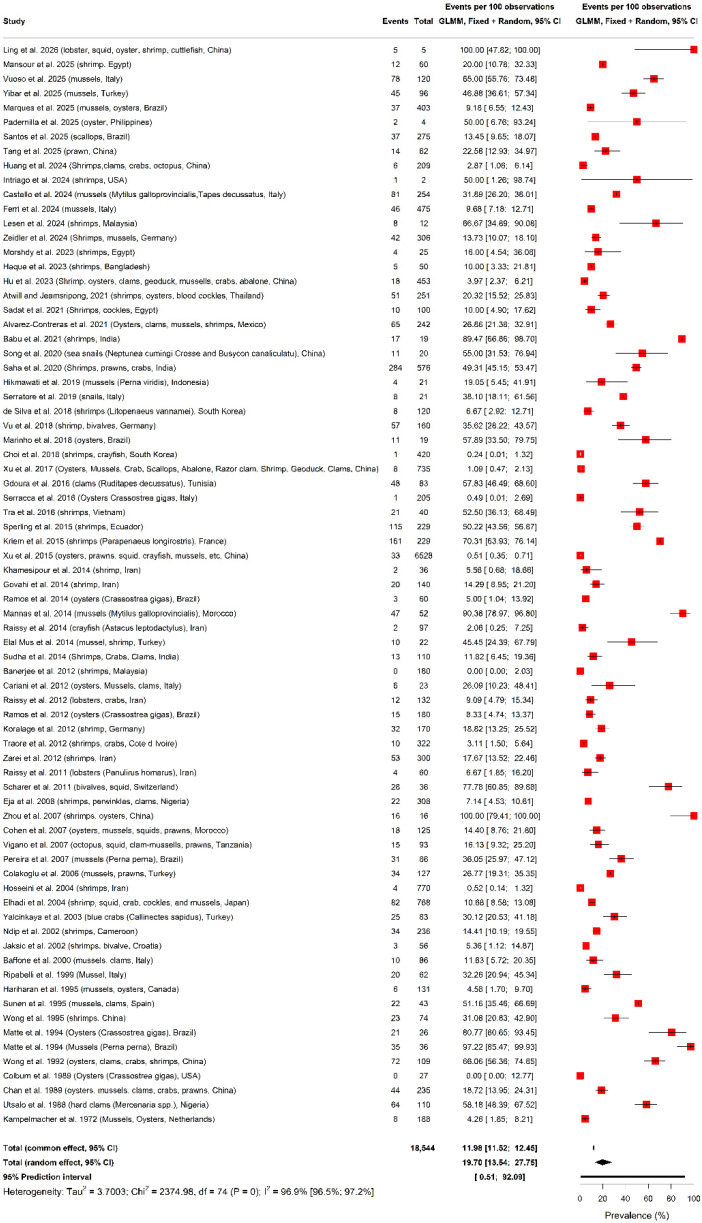
Global pooled prevalence of *V. alginolyticus* contamination in shellfish.

The *V. alginolyticus* global pooled prevalence in shellfish is displayed in Figure 1. *V. alginolyticus* global prevalence was 19.70% (95% confidence intervals (CI): 13.54–27.75, $I^{2}$ = 96.9%, 96.5–97.2, k = 75) in shellfish. While sensitivity analysis based on statistical outlying study removal reduced the global *V. alginolyticus* prevalence in shellfish to 17.50% (14.55–20.89, $I^{2}$ = 77.2%, 68.8–83.3, k = 36), the value ranged from 19.0% (13.0–26.0, $I^{2}$ = 97%) to 21.0% (14.0–29.00, $I^{2}$ = 97%) according to leave-one-out sensitivity analytics (Figure S2a,b). Notably, Egger’s regression intercept test ($\beta_{0}$ = −1.75, −4.23–0.73, *p* = 0.17) confirmed no small-study effects in *V. alginolyticus* pooled global prevalence in shellfish.

### 3.2. Prevalence of V. alginolyticus in Common Shellfish, Species, Class, and Genus

The *V. alginolyticus* pooled prevalence distributed with statistical difference among common types of shellfish ($\chi_{20}^{2}$ = 238.48, *p* < 0.0001; Table 2 and Figure S3a). The snails (50.35%, 1.56–98.48, $I^{2}$ = 88.6%, k = 4) had the highest *V. alginolyticus* prevalence, followed by bivalves (28.97%, 9.47–61.40, $I^{2}$ = 90.8%, k = 5), mussels (20.98%, 10.87–36.62, $I^{2}$ = 94.7%, 24), cephalopods (12.50%, 1.73–53.73, $I^{2}$ = 0.0%, k = 2), oysters (15.16%, 5.66–34.75, $I^{2}$ = 91.5%, k = 21), squid (10.08%, 0.14–89.76, $I^{2}$ = 0.0%, k = 6), cockles (9.40%, 5.59–15.38, $I^{2}$ = 41.7%, k = 4), shrimps (9.24%, 4.95–16.60, $I^{2}$ = 93.0%, k = 48), prawns (8.14%, 1.64–32.03, $I^{2}$ = 70.3%, k = 6), lobsters (6.94%, 1.13–32.68, $I^{2}$ = 0.0%, k = 4), and clams (4.67%, 1.58–13.00, $I^{2}$ = 93.5%, k = 24); crabs (3.11%, 0.66–13.44, $I^{2}$ = 86.4%, k = 14), geoducks (2.23%, 0.43–10.79, $I^{2}$ = 0.0%, k = 3), abalone (1.00%, 0.14–6.75, $I^{2}$ = 0.0%, k = 3), scallops (0.92%, 0.06–11.80, $I^{2}$ = 86.2%, k = 4) and crayfish (0.56%, 0.05–6.16, $I^{2}$ = 0.0%, k = 3) had low pooled rates (Table 2). Figure S3b shows that the *V. alginolyticus* prevalence estimates were robust in oysters ($\beta_{0}$ = −0.578, *p* = 0.71) and mussels ($\beta_{0}$ = −1.312, *p* = 0.48), but indicated the presence of plot asymmetry in shrimps ($\beta_{0}$ = −2.185, *p* = 0.015), clams ($\beta_{0}$ = −4.729, *p* = 0.002), and crabs ($\beta_{0}$ = −3.119, *p* = 0.001).

While pooled *V. alginolyticus* prevalence across shellfish species was statistically different ($\chi_{71}^{2}$ = 435.86, *p* < 0.0001), it registered the highest rate in *Parapenaeus longirostris* (60.96%, 42.19–76.96, $I^{2}$ = 85.5%, k = 2), followed by *Perna perna* (50.98%, 4.71–95.63, $I^{2}$ = 97.1%, k = 3), *Mytilus galloprovincialis* (37.54%, 15.52–66.29, $I^{2}$ = 95.8%, k = 6), *Ruditapes decussatus* (31.93%, 8.18–71.18, $I^{2}$ = 92.0%, k = 2). Other shellfish species such as *Litopenaeus vannamei*, *Crassostrea gigas*, *Loligo* spp., *Penaeus monodon*, *Callinectes sapidus*, *Ruditapes philippinarum*, and *Meretrix lusoria* had a pooled *V. alginolyticus* prevalence of 19.02% (7.54–40.36, $I^{2}$ = 80.30%, k = 11), 15.18% (3.08–50.18, $I^{2}$ = 91.3%, k = 9), 13.16% (7.23–22.76, $I^{2}$ = 0.0%, k = 2), 12.32% (5.32–25.98, $I^{2}$ = 17.6%, k = 4), 11.89% (2.51–41.39, $I^{2}$ = 95.8%, k = 2), 4.91% (0.32–45.71, $I^{2}$ = 90.1%, k = 3), and 0.65% (0.00–99.42, $I^{2}$ = 0.0%, k = 2), respectively (Table 2 and Figure S4).

Generally, the pooled *V. alginolyticus* prevalence declined non-statistically ($\chi_{4}^{2}$ = 7.29, *p* = 0.1214) from 13.37% (0.81–74.36, 0.00% 9) in class *Cephalopoda*, then 10.55% (6.57–16.52, $I^{2}$ = 92.80%, k = 85) in class *Bivalvia*, 6.60% (3.82–11.17, $I^{2}$ = 91.10%, k = 75) in *Malacostraca* to 3.88% (0.19–46.48, $I^{2}$ = 84.40%, k = 9) in class *Gastropoda* (Table 2 and Figure S5a). However, the genus *Parapenaeus*, *Mytilus*, *Perna*, *Litopenaeus Crassostrea*, and *Panulirus* exhibited high pooled *V. alginolyticus* rates of 60.96% (42.19–76.96, $I^{2}$ = 85.50%, k = 2), 37.54% (15.52–66.29%, $I^{2}$ = 95.80%, k = 6), 35.09% (7.84–77.45, $I^{2}$ = 94.10%, k = 5), 19.02% (7.54–40.36, $I^{2}$ = 80.30%, k = 11), 17.50% (4.22–50.51, $I^{2}$ = 90.40%, k = 10), and, 4.47% (0.31–97.11, $I^{2}$ = 0.00%, k = 2), respectively. The pooled *V. alginolyticus* rate ranged from 13.16% (7.23–22.76, $I^{2}$ = 0.00%, k = 2) in the genus *Loligo* to 0.24% (0.00–98.10, $I^{2}$ = 0.00%, k = 3) in the genus *Meretrix*. Notably, *V. alginolyticus* prevalence was statistically different ($\chi_{51}^{2}$ = 414.37, *p* < 0.0001) among the shellfish genera (Table 2 and Figure S6a). While Figure S5b indicates the presence of plot asymmetry in shellfish classes *Malacostraca* (−2.527, *p* = 3.29 × 10^−5^) and *Bivalvia* (−2.355, *p* = 0.002), Figure S6b shows that the *V. alginolyticus* rates are robust in shellfish genera *Crassostrea* ($\beta_{0}$ = −0.061, *p* = 0.98) and *Litopenaeus* ($\beta_{0}$ = −0.824, *p* = 0.63).

### 3.3. Role of Detection Methods in V. alginolyticus Prevalence in Shellfish

The confirmation or detection methods of *V. alginolyticus* play a significant role in its prevalence in shellfish ($\chi_{10}^{2}$ = 150.43, *p* < 0.0001) (Table 2 and Figure S7a). Both targeted MS/culture (k = 5) and qPCR (k = 2) had a detection rate of 100.00% (0.00–100.00, 0.00%). However, MALDI-TOF MS, biochemical test, API, PCR, MALDI-TOF MS/PCR, and mPCR-HPLC recorded pooled *V. alginolyticus* rates of 38.67% (16.14–67.39, $I^{2}$ = 97.90%, k = 3), 20.38% (12.32–31.79, $I^{2}$ = 92.60%, k = 35), 14.44% (7.24–26.74, $I^{2}$ = 85.40%, k = 19), 8.76% (5.63–13.38, $I^{2}$ = 91.20%, k = 87), 3.11% (1.68–5.67, $I^{2}$ = 0.00%, k = 4), and 0.30% (0.14–0.64, $I^{2}$ = 10.10%, k = 20), respectively. The estimate of the *V. alginolyticus* rate in shellfish was robust using API ($\beta_{0}$ = −0.389, *p* = 0.78) and biochemical test ($\beta_{0}$ = −0.754, *p* = 0.62), but PCR ($\beta_{0}$ = −2.541, *p* = 3.61 × 10^−6^), and mPCR-HPLC ($\beta_{0}$ = −1.759, *p* = 6.76 × 10^−5^) indicated the presence of plot asymmetry (Figure S7b).

### 3.4. Distribution of V. alginolyticus in Shellfish Across Nations and Continents

**Figure 2:**
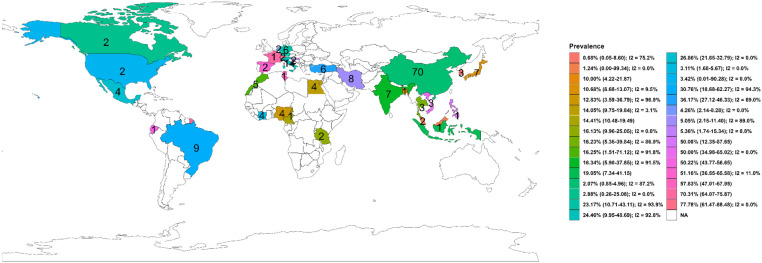
National prevalence of *V. alginolyticus* contamination in shellfish. Numeric values on the map represent amount of disaggregated data from the region.

**Figure 3:**
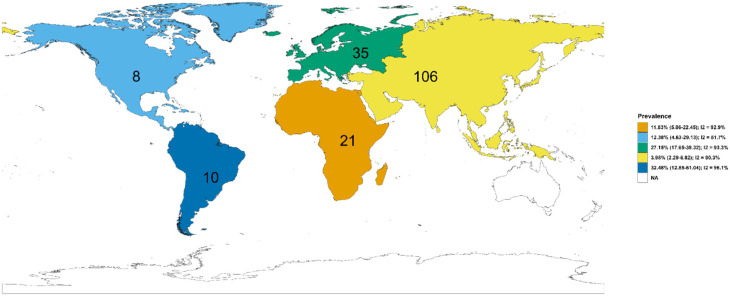
Prevalence of *V. alginolyticus* contamination in shellfish by continent. Numeric values on the map represent amount of disaggregated data from the region.

Figure 2 and Figure 3 detail the distribution of *V. alginolyticus* in shellfish by nation and continent, respectively, which were statistically different (nation: $\chi_{30}^{2}$ = 632.27, *p* < 0.0001; continent: $\chi_{4}^{2}$ = 33.81, *p* < 0.0001). Shellfish-borne *V. alginolyticus* prevalence was descriptively highest in Switzerland with 77.78% (61.47–88.48, $I^{2}$ = 0.00%, k = 2), followed by Spain (51.16%, 36.55–65.58, $I^{2}$ = 11.00%, k = 2), Vietnam (50.00%, 34.98–65.02, $I^{2}$ = 0.00%, k = 3), Turkey (36.17%, 27.12–46.33, $I^{2}$ = 69.00%, k = 6), Brazil (30.76%, 10.68–62.27, $I^{2}$ = 94.30%, k = 9), Mexico (26.86%, 21.65–32.79, $I^{2}$ = 0.00%, k = 4), Italy (24.46%, 9.95–48.69, $I^{2}$ = 92.80%, k = 14), Germany (23.17%, 10.71–43.11, $I^{2}$ = 93.90%, k = 6), India (16.34%, 5.90–37.85, $I^{2}$ = 91.50%, k = 7), Morocco (16.25%, 1.51–71.12, $I^{2}$ = 91.80%, k = 5), Thailand (16.23%, 5.36–39.84, $I^{2}$ = 86.90%, k = 3), Tanzania (16.13%, 9.96–25.05, $I^{2}$ = 0.00%, k = 2), Egypt (14.05%, 9.75–19.84, $I^{2}$ = 3.10%, k = 4), Nigeria (12.83%, 3.59–36.79, $I^{2}$ = 96.90%, k = 4), Japan (10.68%, 8.68–13.07, $I^{2}$ = 9.50%, k = 7), Croatia (5.36%, 1.74–15.34, $I^{2}$ = 0.00%, k = 2), Iran (5.05%, 2.15–11.40, $I^{2}$ = 89.00%, k = 8), The Netherlands (4.26%, 2.14–8.28, $I^{2}$ = 0.00%, k = 2), the USA (3.42%, 0.01–90.28, $I^{2}$ = 0.00%, k = 2), Cote d’Ivoire (3.11%, 1.68–5.67, $I^{2}$ = 0.00%, k = 4), Canada (2.88%, 0.26–25.08, $I^{2}$ = 0.00%, k = 2), China (2.07%, 0.85–4.96, $I^{2}$ = 87.20%, k = 70), Malaysia (1.24%, 0.00–99.34, $I^{2}$ = 0.00%, k = 2), and South Korea (0.68%, 0.05–8.60, $I^{2}$ = 75.20%, k = 3). However, the pooled *V. alginolyticus* rate was 3.98% (2.29–6.82, $I^{2}$ = 90.30%, k = 106), 27.15% (17.65–39.32, $I^{2}$ = 93.30%, k = 35), 11.83% (5.86–22.45, $I^{2}$ = 92.90%, k = 21), 32.48% (12.88–61.04, $I^{2}$ = 96.10%, k = 10), and 12.38% (4.63–29.13, $I^{2}$ = 51.70%, k = 8) in Asian, European, African, South American, and North American shellfish, respectively.

Figure S8 reveals the shellfish-borne *V. alginolyticus* rate showed an absence of plot asymmetry in Italy ($\beta_{0}$ = −0.273, *p* = 0.87) unlike the Chinese funnel plot ($\beta_{0}$ = −1.996, *p* = 0.003) which showed the presence of asymmetry. Similarly, the estimate was robust in Africa ($\beta_{0}$ = −3.287, *p* = 0.09), Europe ($\beta_{0}$ = −0.997, *p* = 0.41), and South America ($\beta_{0}$ = −0.032, *p* = 0.99) in contrast to Asia ($\beta_{0}$ = −2.657, *p* = 5.40 × 10^−8^), which indicated plot asymmetry (Figure S9).

### 3.5. Factors Determining V. alginolyticus Prevalence Estimate in Shellfish

The univariate/bivariate factors affecting the estimate of *V. alginolyticus* prevalence in shellfish is provided in Table 3. Both the additive and multiplicative interactions of nation and sample size explained 73.14% and 58.04% of the true variance in *V. alginolyticus* prevalence in shellfish with interaction weights of $\beta_{0}=-1.89\pm 1.47,\;p=0.199$, $p_{*}$ = 0.001, $F_{31;148}$ = 3.83, *p* = 0.001 and $\beta_{0}=-3.28\pm 3.76,\;p=0.39$, $p_{*}\;(permutation-based\;p-value)$ = 0.001, $F_{54;125}$ = 3.01, *p* = 0.001, respectively. Other univariate factors include confirmation techniques ($R^{2}$ = 46.59%, $\beta_{0}=-1.63\pm 0.83,\;p=0.05$, $p_{*}$ = 0.001, $F_{10;169}$ = 11.16, *p* = 0.001), nation ($R^{2}$ = 37.72% −2.20±1.74, p=0.21, $p_{*}$ = 0.006, $F_{30;149}$ = 1.89, *p* = 0.006), medium ($R^{2}$ = 19.42%, 2.24±1.85, p=0.23, $p_{*}$ = 0.031, $F_{15;164}$ = 1.69, *p* = 0.03), shellfish type ($R^{2}$ = 16.99% −3.90±1.32, p=0.004, $p_{*}$ = 0.122, $F_{20;159}$ = 1.34, *p* = 0.12), and continent ($R^{2}$ = 15.29%, $-1.81\pm 0.41,\;p=1.57\times 10^{-5}$, $p_{*}$ = 0.001, $F_{4;175}$ = 5.90, *p* = 0.001). Particularly, bivalves (β=3.06±1.57, *p* = 0.05), mPCR-HPLC (β=−3.65±0.92, *p* = 0.0001), qPCR (β=4.46±1.67, *p* = 0.008), and targeted MS/culture (β=2.73±1.28, *p* = 0.04) were detection methods that significantly influenced the *V. alginolyticus* pooled estimate. Sample size also had an indirect relationship ($\beta =-0.01\pm 0.00,\;p=6.25\times 10^{-11}$).

## 4. Discussion

*Vibrio alginolyticus* is a halophilic bacterium naturally found in warm waters and marine environments, and has been implicated in seafood-borne illness [1,2]. The US CDC notes that *V. alginolyticus* is one of the three *Vibrio* species along with *V. parahaemolyticus* and *V. vulnificus* that cause human vibriosis [22]. From 1988 to 2012 for instance, the reported *V. alginolyticus* infections in the USA increased by 12-fold, 96% of which were from coastal states and 86% involved in water activities [96]. Raw/undercooked shellfish is the common transmission vehicle for *Vibrio*, and global vibriosis incidence is substantial at tens of thousands of annual cases in the US alone [22]. The current study shows that *V. alginolyticus* is widespread in shellfish and its global pooled rate is 19.7% (95%, 13.5–27.8). This rate is comparable to published *V. parahaemolyticus* prevalence estimates in seafood [97], underlining that *V. alginolyticus* is common in shellfish. However, the prevalence is accompanied by a high heterogeneity ($I^{2}$ = 97%) which reflects the wide variation in *V. alginolyticus* estimates arising from methodological differences across the included studies and regions. Despite this variability, the *V. alginolyticus* prevalence data from shellfish signal a clear public health concern; even though *V. alginolyticus* infections are typically less severe than *V. vulnificus*, they can cause gastroenteritis, otitis and wound infections, especially in susceptible groups like the immunocompromised, immunosuppressed, infants, and the elderly [22,98]. Furthermore, climate change is expected to expand *Vibrio* habitats, since warmer, low-salinity waters promote *Vibrio* proliferation [97], suggesting that risks from all pathogenic *Vibrio* spp. including *V. alginolyticus* will increase in future.

The findings from this study have important regulatory and surveillance implications of *V. alginolyticus* for shellfish food safety. At present, international standards for shellfish/seafood safety focus on *V. parahaemolyticus* and *V. vulnificus*. *Codex Alimentarius*’s existing guidelines (CAC/GL 73–2010) address “pathogenic *Vibrio*” but historically emphasized *V. parahaemolyticus* and *V. vulnificus* [96,99,100,101]. However, recent *Codex* deliberations recognize the need to update seafood/shellfish safety guidance to include *V. alginolyticus* (and *V. cholerae*) and to broaden its coverage beyond molluscan shellfish to other shellfish groups [102]. Similarly, in 2016 the FAO/WHO issued guidance and emphasized PCR and other molecular tests as the reliable detection methods for *Vibrio* spp. in seafood [103]. Currently, the EU has no specific microbiological limits for *V. alginolyticus* in shellfish/seafood (Regulation 2073/2005 sets no *Vibrio* limits) [97,104]. In addition, a 2024 BIOHAZ EFSA opinion report treated *V. alginolyticus* as a “minor” concern in shellfish/seafood compared to *V. parahaemolyticus*/*vulnificus* [97] but did highlight *V. alginolyticus* knowledge gaps in seafood and anticipated rising levels of *Vibrio* including *V. alginolyticus* under warming climates in seafood/shellfish [97]. In the US, FDA seafood controls concentrate on *V. parahaemolyticus* and *V. vulnificus* [105], with no explicit *V. alginolyticus* criteria. In practice, harmonizing *V. alginolyticus* standards and microbiological criteria in shellfish/seafood is hampered by scarce data and surveillance gaps. Only a few countries (notably the USA) have systematic *Vibrio* monitoring in seafood/shellfish, and many regions lack any routine screening of *Vibrio* and *V. alginolyticus* in shellfish [19,98]. For example, our study found few studies from Africa and Latin America and none from Oceania that screened *V. alginolyticus* in shellfish, meaning the real *V. alginolyticus* burden in shellfish is underestimated [19,98]. Even in countries where *Vibrio* is well-studied/screened in shellfish/seafood, surveillance often misses non-cholera *Vibrios*, suggesting an urgent need for coordinated international monitoring of *Vibrio* including *V. alginolyticus* in shellfish (*Codex* and the WHO are planning new risk assessments and guidelines that include *V. alginolyticus*) [102,105].

Geographically and by species, the results from this study reveal striking patterns in *V. alginolyticus* prevalence in shellfish. *V. alginolyticus*-contaminated shellfish were detected worldwide, but its prevalence differed. Europe and South America showed the highest pooled *V. alginolyticus* prevalence rates (27% and 32%, respectively, across studies) in shellfish, while aggregated shellfish data from Asia showed a surprisingly low *V. alginolyticus* rate (~4%). This low pooled *V. alginolyticus* prevalence in Asian shellfish was driven by many Chinese studies (pooled 2% *V. alginolyticus* prevalence), whereas smaller datasets from Vietnam, Thailand and India showed higher pooled *V. alginolyticus* rates (16–50%) (Table 1). This could be attributed to the use of suboptimal detection methods such as mPCR-HPLC in China that yielded a close-to-zero *V. alginolyticus* positivity rate despite large shellfish samples (6528) being tested. In Africa, only a handful of shellfish were screened for *V. alginolyticus*, and small studies exist (e.g., Nigeria 13%, Morocco 16% pooled *V. alginolyticus* rates, respectively), while there were none from major producers like Australia, Japan, or New Zealand. The highest country-level *V. alginolyticus* prevalence in shellfish was found in Switzerland (78%) and Spain (51%), albeit from limited shellfish samples/studies, reflecting outbreaks or targeted surveillance. By shellfish type, gastropods (snails) topped the list with a 50% pooled *V. alginolyticus* rate, followed by bivalves in general (29%) and mussels (21%). Among *P. longirostris*, *R. decussatus*, *P. perna*, *M. galloprovincialis*, *R. decussatus*, *Loligo* spp., *P. monodon*, *C. sapidus*, and *R. philippinarum*, the pooled *V. alginolyticus* positivity declined from 67% to 5% (k ≤ 6), but with a more stable prevalence in *L. vannamei* (19.02%, k = 11) and *C. gigas* (15.18%, k = 9). Edible oysters and clams had lower *V. alginolyticus* pooled rates (4–15%) in shellfish, but given their global consumption, these moderate rates still imply sizeable *V. alginolyticus* risk in molluscan shellfish. In contrast, crabs, scallops, and other shellfish had only a few percent prevalence. These patterns likely reflect feeding habitats, harvesting practices and host specificity. For example, filter-feeding bivalves concentrate *Vibrio* more efficiently than detritus-feeding crustaceans, but the data also suffers from heterogeneity.

The choice of laboratory method influenced reported *V. alginolyticus* prevalence in shellfish. Studies using extremely sensitive or specific assays found *V. alginolyticus* in many shellfish assayed. For example, targeted culture with MALDI-TOF or species-specific qPCR detected *V. alginolyticus* in 100% of tested shellfish samples. This, however, reflects the extreme sensitivity of qPCR/MALDI-TOF in detecting *V. alginolyticus* in shellfish rather than ubiquitous *V. alginolyticus* contamination in every single shellfish on earth.

By contrast, conventional phenotypic tests detected much less *V. alginolyticus* in shellfish yielding a pooled prevalence of 20% by general biochemical/API tests, 8.8% by generic PCR, and near-zero (0.3–3%) by multiplex PCR-HPLC or MALDI-TOF/PCR in our analysis. This discrepancy (statistically significant, *p* < 0.0001) implies that many studies likely underestimated *V. alginolyticus* in sampled shellfish when using routine methods. In addition, methods such as multiplex PCR-HPLC or MALDI-TOF/PCR that relied on culture might be undermined/limited in sensitivity by viable-but-nonculturable *V. alginolyticus* cells. It highlights a key bias that *V. alginolyticus* prevalence figures in included data sources depend on analytical sensitivity. Regulatory monitoring of *V. alginolyticus* in shellfish that relies on older culture/biochemical protocols may thus miss contaminated batches. Standardizing and updating detection methods (e.g., implementing qPCR or validated MALDI-TOF workflows) would improve the accuracy of *V. alginolyticus* surveillance in shellfish. These challenges underline the need for harmonized *V. alginolyticus* monitoring in shellfish and modern detection techniques. Standardized *V. alginolyticus* surveillance protocols in shellfish (ideally validated molecular methods) should be adopted internationally. Not surprisingly, the FAO/WHO 2016 guidance advocates the use of PCR and genomic methods for reliable enumeration of *Vibrio* in seafood [103]. EU and US labs are increasingly turning to MALDI-TOF and whole-genome sequencing for *Vibrio* identification especially in seafood [99], and similar approaches are encouraged for *V. alginolyticus*. A pan-European ring trial showed that standardized ISO methods can improve consistency of *Vibrio* surveillance in seafood [103]. Harmonized one-health frameworks for *V. alginolyticus* (as recommended for antibiotic-resistant *Vibrio*) [98] would align clinical, environmental and food-borne surveillance of *V. alginolyticus* using source-tracking and molecular profiling techniques, and may offer a clue to *V. alginolyticus*’s definitive roles in seafood- or shellfish-borne illness. Given the global trade in shellfish, international data sharing is also critical. Currently, surveillance gaps and a lack of regulatory criteria for *V. alginolyticus* mean potential hazards may be missed. For example, one recent WHO *Codex* review noted little information on *V. alginolyticus* in seafood and called for JEMRA to assess it [102].

The strengths of the current study include its broad scope and quantitative synthesis of the largest diverse global dataset on *V. alginolyticus* in shellfish to date. Sensitivity checks showed the pooled *V. alginolyticus* prevalence estimate (~18–21%) in shellfish to be stable, and Egger’s test found no evidence of small-study publication bias (*p* = 0.17). Nonetheless, the findings have important caveats and limitations. Heterogeneity was high (global $I^{2}$ = 96–97%), indicating that datasets were from diverse environments, host species, sample handling and methods; thus, the pooled *V. alginolyticus* prevalence in shellfish should be interpreted cautiously. Many subgroup estimates of *V. alginolyticus* prevalence in shellfish are based on few studies (some species or countries have k < 5), yielding wide confidence bounds and potential random error/undermining precision. The overall prevalence does not distinguish pathogenic versus avirulent strains of *V. alginolyticus*, so it does not imply direct disease risk. Crucially, detection methods varied widely and strongly affected pooled *V. alginolyticus* prevalence results in shellfish. Targeted enrichment or qPCR assays tended to find much higher prevalence (even 100% in small trials) than conventional culture or biochemical kits, suggesting either false positives by culture or false negatives by some assays. These discrepancies imply that under-standardized methods could under- or overestimate true *V. alginolyticus* levels in shellfish. Viable-but-nonculturable cells and strain-level differences further complicate the *V. alginolyticus* detection rate in shellfish. Thus, one limitation of the aggregated results is methodological inconsistency: pooling PCR and culture data assumes comparability that may not hold. Nonetheless, our meta-regression found that both the nation of sampling and sample size were major sources of variance (together explaining >70% of heterogeneity), implying that systematic factors (geography, study design) drive most differences.

## 5. Conclusions and Recommendations

In summary, *V. alginolyticus* is a widespread shellfish contaminant that carries real public health implications. Our study reveals substantial occurrence of *V. alginolyticus* in shellfish worldwide, with notable regional and species-specific hotspots. *V. alginolyticus*’s potential public health risk is heightened by its presence in commonly eaten shellfish and by its incomplete monitoring in shellfish safety programs. Considering the high prevalence of *V. alginolyticus* in shellfish and its established pathogenicity, existing shellfish safety guidelines should be updated to explicitly include *V. alginolyticus*, and more countries need to implement its routine testing (as part of *Codex*/WHO/FAO frameworks) in the interim as precautionary controls to protect shellfish consumers. It is recommended that standardized ISO/PCR protocols should be applied in *V. alginolyticus* monitoring in shellfish for better performance. In addition, future work should synthesize or perform meta-analysis of data related to the *V. alginolyticus* virulence gene (e.g., *vhvp*, hemolysins) in shellfish isolates. At the same time, the limitations of past studies (heterogeneity, small samples, and variable methods) mean results should be read with caution. Ultimately, harmonized and molecular-based surveillance of *V. alginolyticus* in shellfish linked to food safety criteria will be essential to manage the emerging threat of *V. alginolyticus* in shellfish.

## Figures and Tables

**Table 1:** Overview of *V. alginolyticus* contamination in shellfish.

Author	Year	N	P	Sample Type	Species	Confirmation	Medium	Nation	Continent
[4]	2026	5	5	lobster, squid, oyster, shrimp, cuttlefish		targeted MS/culture		China	Asia
[5]	2025	60	12	shrimp	*Litopenaeus vannamei*	PCR	TCBS	Egypt	Africa
[6]	2025	120	78	mussels		MALDI-TOF/MS	ISO 21872-1:2017	Italy	Europe
[7]	2025	96	45	mussels		MALDI-TOF/MS	TCBS	Turkey	Europe
[8]	2025	403	37	mussels, oysters	*Perna perna*/*Ostreidae*	PCR	TCBS	Brazil	South America
[9]	2025	4	2	oyster	*Magallana bilineata*	Seq	TCBS	Philippines	Asia
[10]	2025	275	37	scallops	*Nodipecten nodosus*	MALDI-TOF/MS	BHI	Brazil	South America
[11]	2025	62	14	prawn	*Macrobrachium rosenbergii*	PCR	TCBS	China	Asia
[32]	2024	209	6	shrimps, clams, crabs, octopus	*Parapenaeopsis hardwickii*, *Trachypenaeus curvirostris*, *Penaeus japonicus*, *Fenneropenaeus chinensis*, *Exopalaemon carinicauda*, *Oratosquilla oratoria*, *Palaemon gravieri*	PCR	TCBS	China	Asia
[33]	2024	2	1	shrimps	*Penaeus vannamei*	PCR	TCBS/CHROMagar	USA	North America
[34]	2024	254	81	mussels, clams		PCR	TCBS/CHROMagar	Italy	Europe
[35]	2024	475	46	mussels	*Mytilus galloprovincialis*	PCR	TCBS	Italy	Europe
[36]	2024	12	8	shrimps	*Litopenaeus vannamei*	PCR	APW	Malaysia	Asia
[17]	2024	306	42	shrimps, mussels	*Penaeus monodon*, *Litopenaeus vannamei*, *Pleoticus muelleri*	PCR	TCBS/Vibrio ChromoSelect Agar	Germany	Europe
[37]	2023	25	4	shrimps		PCR	TCBS	Egypt	Africa
[38]	2023	50	5	shrimps	Shrimp, crab, squid, *Logio* sp., *Spia* sp., *Anadara granosa*, *Lithophaga malaccana*, mussels	PCR	TCBS	Bangladesh	Asia
[39]	2023	453	18	shrimp, oysters, clams, geoduck, mussels, crabs, abalone		PCR	APW	China	Asia
[40]	2021	251	51	shrimps, oysters, blood cockles			TCBS/CHROMagar	Thailand	Asia
[41]	2021	100	10	shrimps, cockles		PCR	TCBS	Egypt	Africa
[21]	2021	242	65	oysters, clams, mussels, shrimps		PCR	TCBS	Mexico	North America
[42]	2021	19	17	shrimps	*Litopenaeus vannamei*, *Penaeus monodon*	PCR	TCBS	India	Asia
[43]	2020	20	11	sea snails	*Neptunea cumingi* Crosse, *Busycon canaliculatu*	VITEK	TCBS	China	Asia
[44]	2020	576	284	shrimps, prawns, crabs		PCR	TCBS	India	Asia
[45]	2019	21	4	mussels	*Perna viridis*	PCR	TCBS	Indonesia	Asia
[46]	2019	21	8	snails		PCR	TCBS/CHROMagar Vibrio	Italy	Europe
[47]	2018	120	8	shrimps	*Litopenaeus vannamei*	PCR	TCBS	South Korea	Asia
[48]	2018	160	57	shrimp, bivalves	white leg shrimp, black tiger shrimp, blue mussels, Venus clams, razor shells, and cockles	PCR	ISO/TS 21872	Germany	Europe
[49]	2018	19	11	oysters		Bioch	TCBS	Brazil	South America
[50]	2018	420	1	shrimps, crayfish		PCR	TCBS	South Korea	Asia
[51]	2017	735	8	oysters, mussels, crab, scallops, abalone, razor clam, shrimp, geoduck, clams		PCR		China	Asia
[52]	2016	83	48	clams	*Ruditapes decussatus*	PCR	ISO/TS 21872-1/TCBS	Tunisia	Africa
[53]	2016	205	1	oysters	*Crassostrea gigas*	PCR	ISO/TS 21872-1	Italy	Europe
[54]	2016	40	21	shrimps	*Litopenaeus vannamei*, *Penaeus monodon*, *Penaeus merguiensis*, *Metapenaeus ensis*, *Macrobrachium rosenbergii*	PCR	TCBS	Vietnam	Asia
[55]	2015	229	115	shrimps		PCR	ISO/TS 21872-2:2007/TCBS	Ecuador	South America
[56]	2015	229	161	shrimps	*Parapenaeus longirostris*	PCR	TCBS/CHROMagar Vibrio	France	Europe
[57]	2015	6528	33	oysters, prawns, squid, crayfish, mussels, scallops, *Chlamys farreri*, crab, razor clam, *Bullacta exarata*, shrimp, *R. philippinarum*, *Aplysia*, abalone, geoduck, *Scapharca subcrenata*, lobster, clams, Corbicula leana, poker-chip Venus, China		mPCR-HPLC	TCBS	China	Asia
[58]	2014	36	2	shrimp		PCR	TCBS	Iran	Asia
[59]	2014	140	20	shrimp	*Litopenaeus vannamei*	PCR		Iran	Asia
[60]	2014	60	3	oysters	*Crassostrea gigas*	API	TCBS	Brazil	South America
[61]	2014	52	47	mussels	*Mytilus galloprovincialis*	API 20 E	ISO/TS 16649-3 (2005)/TCBS/CHROMagar Vibrio	Morocco	Africa
[62]	2014	97	2	crayfish	*Astacus leptodactylus*	PCR	TCBS	Iran	Asia
[63]	2014	22	10	mussel, shrimp		API 20E	TCBS	Turkey	Europe
[64]	2014	110	13	shrimps, crabs, clams	*Penaeus indicus*, *Penaeus monodon*, *Metapenaeus dobsonii*, *Metapenaeus affinis*, *Parapenaeopsis stylifera*; *Scylla serrata*, *Portunus pelagicus*, *Charybdis crussiata*, *Villorita cyprinoides*	Bioch	TCBS	India	Asia
[65]	2012	180	0	shrimps	*Litopenaeus vannamei*	API 20E	TCBS	Malaysia	Asia
[66]	2012	23	6	oysters, mussels, clams		PCR	TCBS	Italy	Europe
[67]	2012	132	12	lobsters, crabs		PCR	TCBS/mCPC	Iran	Asia
[68]	2012	180	15	oysters	*Crassostrea gigas*	API 20E	TCBS	Brazil	South America
[69]	2012	170	32	shrimp	*Penaeus monodon*	PCR	TCBS	Germany	Europe
[70]	2012	322	10	shrimps, crabs	*Penaeus*, *Macrobrachium*, *Callinectes*, *Cardisoma*	MALDI-TOF/PCR	TCBS	Cote d’Ivoire	Africa
[71]	2012	300	53	shrimps		PCR	TCBS	Iran	Asia
[72]	2011	60	4	lobsters	*Panulirus homarus*	PCR	TCBS	Iran	Asia
[73]	2011	36	28	bivalves, squid		PCR	TCBS	Switzerland	Europe
[74]	2008	308	22	shrimps, periwinkles, clams		Bioch	TCBS	Nigeria	Africa
[75]	2007	16	16	shrimps, oysters		qPCR	TCBS	China	Asia
[76]	2007	125	18	oysters, mussels, squid, prawns		PCR	TCBS	Morocco	Africa
[77]	2007	93	15	octopus, squid, clam–mussels, prawns		API-20E	TCBS	Tanzania	Africa
[78]	2007	86	31	mussels	*Perna perna*	Bioch	TCBS	Brazil	South America
[79]	2006	127	34	mussels, prawns	*Mytilus galloprovincialis*, *Donax trunculus*, *Parapenaus longirostris*	API/Bioch	TCBS	Turkey	Europe
[80]	2004	770	4	shrimps		Bioch	TCBS	Iran	Asia
[81]	2004	768	82	shrimp, squid, crab, cockles, and mussels	Shrimp, crab, squid, *Logio* sp., *Spia* sp., *Anadara granosa*, *Lithophaga malaccana*, mussels	Bioch	TCBS	Japan	Asia
[82]	2003	83	25	blue crabs	*Callinectes sapidus*	Bioch	TCBS	Turkey	Europe
[83]	2002	236	34	shrimps	*Macrobranchium* spp.	API 20E	TCBS	Cameroon	Africa
[84]	2002	56	3	shrimps, bivalves		API 20E	TCBS	Croatia	Europe
[85]	2000	86	10	mussels, clams	*Mytilus galloprovincialis*, *Venus gallina*	Bioch	TCBS	Italy	Europe
[86]	1999	62	20	mussels	*Mytilus galloprovincialis*	Bioch	TCBS	Italy	Europe
[87]	1995	131	6	mussels, oysters		Bioch	TCBS	Canada	North America
[88]	1995	43	22	mussels, clams		Bioch	TCBS	Spain	Europe
[89]	1995	74	23	shrimps		Bioch	TCBS	China	Asia
[90]	1994	26	21	oysters	*Crassostrea gigas*	Bioch	TCBS	Brazil	South America
[90]	1994	36	35	mussels	*Perna perna*	Bioch	TCBS	Brazil	South America
[91]	1992	109	72	oysters, clams, crabs, shrimps		Bioch	TCBS	China	Asia
[92]	1989	27	0	oysters	*Crassostrea gigas*	API 20E	TCBS	USA	North America
[93]	1989	235	44	oysters, mussels, clams, crabs, prawns		API 20E	TCBS	China	Asia
[94]	1988	110	64	hard clams	*Mercenaria* spp.	Bioch	TCBS	Nigeria	Africa
[95]	1972	188	8	mussels, oysters		Bioch	TCBS	Netherlands	Europe

Bioch = biochemical test; TCBS = thiosulfate–citrate–bile salts–sucrose agar; Seq = sequencing; PCR = polymerase chain reaction; APW = alkaline peptone water; API = analytical profile index; N = sample size; P = positive.

**Table 2:** *V. alginolyticus* prevalence in shellfish by subgroups.

Subgroup	Prevalence (95 CI)	I^2^	k
$\mathrm{Nation}\;(\chi_{30}^{2}$ = 632.27, *p* < 0.0001)			
$\mathrm{Species}\;(\chi_{71}^{2}$ = 435.86, *p* < 0.0001)			
Nrp	5.51% (3.02–9.82)	93.70%	72
*Crassostrea gigas*	15.18% (3.08–50.18)	91.30%	9
*Litopenaeus vannamei*	19.02% (7.54–40.36)	80.30%	11
*Mytilus galloprovincialis*	37.54% (15.52–66.29)	95.80%	6
*Penaeus monodon*	12.32% (5.32–25.98)	17.60%	4
*Perna perna*	50.98% (4.71–95.63)	97.10%	3
*Ruditapes philippinarum*	4.91% (0.32–45.71)	90.10%	3
*Loligo* spp.	13.16% (7.23–22.76)	0.00%	2
*Ruditapes decussatus*	31.93% (8.18–71.18)	92.00%	2
*Parapenaeus longirostris*	60.96% (42.19–76.96)	85.50%	2
*Perna viridis*	19.72% (12.04–30.59)	0	2
*Fenneropenaeus chinensis*	1.78% (0.07–31.48)	0	2
*Meretrix lusoria*	0.65% (0.00–99.42)	0.00%	2
*Callinectes sapidus*	11.89% (2.51–41.39)	95.80%	2
$\mathrm{Shellfish}\;\mathrm{type}\;(\chi_{20}^{2}$ = 238.48, *p* < 0.0001)			
Shrimps	9.24% (4.95–16.60)	93.00%	48
Mussels	20.98% (10.87–36.62)	94.70%	24
Clams	4.67% (1.58–13.00)	93.50%	24
Oysters	15.16% (5.66–34.75)	91.50%	21
Crabs	3.11% (0.66–13.44)	86.40%	14
Squid	10.08% (0.14–89.76)	0.00%	6
Prawns	8.14% (1.64–32.03)	70.30%	6
Bivalves	28.97% (9.47–61.40)	90.80%	5
Lobsters	6.94% (1.13–32.68)	0.00%	4
Scallops	0.92% (0.06–11.80)	86.20%	4
Cockles	9.40% (5.59–15.38)	41.70%	4
Snails	50.35% (1.56–98.48)	88.60%	4
Abalone	1.00% (0.14–6.75)	0.00%	3
Geoducks	2.23% (0.43–10.79)	0.00%	3
Crayfish	0.56% (0.05–6.16)	0.00%	3
Cephalopods	12.50% (1.73–53.73)	0.00%	2
$\mathrm{Confirmation}\;(\chi_{10}^{2}$ = 150.43, *p* < 0.0001)			
PCR	8.76% (5.63–13.38)	91.20%	87
Biochemical test	20.38% (12.32–31.79)	92.60%	35
mPCR-HPLC	0.30% (0.14–0.64)	10.10%	20
API	14.44% (7.24–26.74)	85.40%	19
Targeted MS/culture	100.00% (0.00–100.00)	0.00%	5
MALDI-TOF MS/PCR	3.11% (1.68–5.67)	0.00%	4
MALDI-TOF MS	38.67% (16.14–67.39)	97.90%	3
Nrp	16.23% (5.36–39.84)	86.90%	3
qPCR	100.00% (0.00–100.00)	0.00%	2
$\mathrm{Continent}\;(\chi_{4}^{2}$ = 33.81, *p* < 0.0001)			
$\mathrm{Class}\;(\chi_{4}^{2}$ = 7.29, *p* = 0.1214)			
*Bivalvia*	10.55% (6.57–16.52)	92.80%	85
*Malacostraca*	6.60% (3.82–11.17)	91.10%	75
*Cephalopoda*	13.37% (0.81–74.36)	0.00%	9
*Gastropoda*	3.88% (0.19–46.48)	84.40%	9
Nrp	33.05% (11.06–66.23)	97.30%	2
$\mathrm{Genus}\;(\chi_{49}^{2}$ = 244.79, *p* < 0.0001)			
Nrp	6.00% (3.41–10.34)	92.80%	78
*Crassostrea*	17.50% (4.22–50.51)	90.40%	10
*Penaeus*	13.03% (3.88–35.75)	87.50%	10
*Litopenaeus*	19.02% (7.54–40.36)	80.3%	11
*Mytilus*	37.54% (15.52–66.29)	95.80%	6
*Perna*	35.09% (7.84–77.45)	94.10%	5
*Ruditapes*	11.16% (1.70–47.77)	92.70%	5
*Macrobrachium*	7.47% (1.43–31.00)	60.30%	3
*Meretrix*	0.24% (0.00–98.10)	0.00%	3
*Panulirus*	24.47% (0.31–97.11)	0.00%	2
*Loligo*	13.16% (7.23–22.76)	0.00%	2
*Portunus*	8.73% (0.03–97.08)	0.00%	2
*Scylla*	8.33% (3.16–20.19)	0.00%	2
*Parapenaeus*	60.96% (42.19–76.96)	85.50%	2
*Corbicula*	4.23% (0.03–85.54)	97.00%	2
*Scapharca*	0.41% (0.00–81.38)	0.00%	2
*Callinectes*	11.89% (2.51–41.39)	95.80%	2

Nrp = not reported.

**Table 3:** Factors determining the prevalence estimate of *V. alginolyticus* in shellfish.

Model	*R* ^2^	$\hat{\beta_{0}}\;\pm \;SE$, *p* Value	*p* Value	Test of Moderators
Nation ∗ N	73.14%	−3.28±3.76, p=0.39	0.001	$F_{54;125}$ = 3.01, *p* = 0.001
Nation + N	58.04%	−1.89±1.47, p=0.199	0.001	$F_{31;148}$ = 3.83, *p* = 0.001
Confirmation	46.59%	−1.63±0.83, p=0.05	0.001	$F_{10;169}$ = 11.16, *p* = 0.001
Nation	37.72%	−2.20±1.74, p=0.21	0.006	$F_{30;149}$ = 1.89, *p* = 0.006
Medium	19.42%	2.24±1.85, p=0.23	0.031	$F_{15;164}$ = 1.69, *p* = 0.03
Shellfish type	16.99%	−3.90±1.32, p=0.004	0.122	$F_{20;159}$ = 1.34, *p* = 0.12
Continent	15.29%	$-1.81\pm 0.41,\;p=1.57\times 10^{-5}$	0.001	$F_{4;175}$ = 5.90, *p* = 0.001
Year	1.76%	39.04±27.79, p=0.16	0.134	$F_{1;178}$ = 2.18, *p* = 0.13
Class	1.08%	−0.72±1.34, p=0.59	0.744	$F_{4;175}$ = 0.44, *p* = 0.74

$\hat{\beta_{0}}\pm SE$ = predicted effect size when x = 0 (regression coefficient)/standard error; $R^{2}$ = coefficient of determination; df1 and df2 are degrees of freedoms.

## Data Availability

The original contributions presented in this study are included in the article/Supplementary Materials. Further inquiries can be directed to the corresponding authors.

## Supplementary Materials

The following supporting information can be downloaded at: https://www.mdpi.com/article/10.3390/foods15101642/s1, Figure S1. Flow diagram for processing data sources on shellfish-borne *V. alginolyticus*; Figure S2a: Sensitivity analysis of prevalence of *V. alginolyticus* contamination in shellfish based on outliers removal; Figure S2b: Leave-out-sensitivity analysis outcomes of *V. alginolyticus* contamination prevalence in shellfish; Figure S3a: Prevalence of *V. alginolyticus* in different shellfish types; Figure S3b: Subgroup funnel plot for *V. alginolyticus* prevalence in different shellfish types; Figure S4: Prevalence of *V. alginolyticus* in different shellfish species; Figure S5a: Prevalence of *V. alginolyticus* in different shellfish classes; Figure S5b: Subgroup funnel plot for prevalence of *V. alginolyticus* in different shellfish; Figure S6a: Prevalence of *V. alginolyticus* in different shellfish genera; Figure S6b: Subgroup funnel plot for prevalence of *V. alginolyticus* in different shellfish genera; Figure S7a: Prevalence of *V. alginolyticus* in different shellfish by detection assays; Figure S7b: Subgroup funnel plot for prevalence of *V. alginolyticus* in shellfish by detection assays; Figure S8. Subgroup funnel plot for prevalence of *V. alginolyticus* by nation; Figure S9: Subgroup funnel plot for prevalence of *V. alginolyticus* in shellfish by continent; Table S1: Disaggregated dataset for subgroup analysis.
