# Supplementary material for: Occurrence and Distribution of Vibrio alginolyticus in Shellfish
Source: Foods. 2026 May 8;15(10):1642. doi: 10.3390/foods15101642 (PMC13205350; doi:10.3390/foods15101642)
Supplement: Supplementary file 1 [file foods-15-01642-s001.zip › foods-4265123-supplementary.pdf]

Supplementary materials for "Occurrence and distribution of *Vibrio alginolyticus* in shellfish"

Temitope C. Ekundayo* and Frederick T. Tabit
Department of Life and Consumer Sciences, University of South Africa,Cnr Christiaan de Wet and Pioneer Ave, Florida, Private Bag X6, 1710, Roodepoort, South Africa
*Corresponding author email cyruscyrusthem@gmail.com

Supplementary methods

Detail search strategies

Web of Science

TS=(alginolyticus AND (seafood* OR shellfish* OR bivalve* OR crayfish* OR crawfish* OR oyster* OR mussel* OR clams OR cockle* OR scallop* OR abalone* OR conch* OR limpet* OR whelk* OR periwinkle* OR shrimp* OR mollus* OR prawn* OR crab* OR lobster* OR krill* OR gooseneck* OR barnacle* OR urchin OR jellyfish* OR cattail* OR octopus* OR squid* OR cuttlefish*)) AND DT=(Article)

Date Run: Wed Aug 27 2025 10:42:15 GMT+0200 (South Africa Standard Time)

Scopus

(TITLE-ABS-KEY ((alginolyticus AND (seafood* OR shellfish* OR bivalve* OR crayfish* OR crawfish* OR oyster* OR mussel* OR clams OR cockle* OR scallops OR abalone* OR conch* OR limpet* OR whelk* OR periwinkle* OR shrimp* OR mollus* OR prawn* OR crab* OR lobster* OR krill* OR gooseneck* OR barnacle* OR urchin OR jellyfish* OR cattail* OR octopus* OR squid* OR cuttlefish*))) AND (LIMIT-TO (DOCTYPE,"ar")))

Date Run: 2025/08/27

PubMed

alginolyticus[tiab] AND (seafood*[tiab] OR shellfish*[tiab] OR crayfish*[tiab] OR crawfish*[tiab] OR bivalve*[tiab] OR oyster*[tiab] OR mussel*[tiab] OR clams[tiab] OR cockle*[tiab] OR scallops[tiab] OR abalone*[tiab] OR conch*[tiab] OR limpet*[tiab] OR whelk*[tiab] OR periwinkle*[tiab] OR shrimp*[tiab] OR mollus*[tiab] OR prawn*[tiab] OR crab*[tiab] OR lobster*[tiab] OR krill*[tiab] OR gooseneck*[tiab] OR barnacle*[tiab] OR 'sea urchin'[tiab] OR jellyfish*[tiab] OR cattail*[tiab] OR octopus*[tiab] OR squid*[tiab] OR cuttlefish*[tiab]) NOT (review[Publication Type] OR systematic review[Publication Type] OR meta-analysis[Publication Type] OR retracted publication[Publication Type])

Date Run: 2025/08/27; Time: 04:51:34

Ruditapes philippinarum = Tapes philippinarum
Tapes decussatus = Ruditapes decussatus

Supplementary results

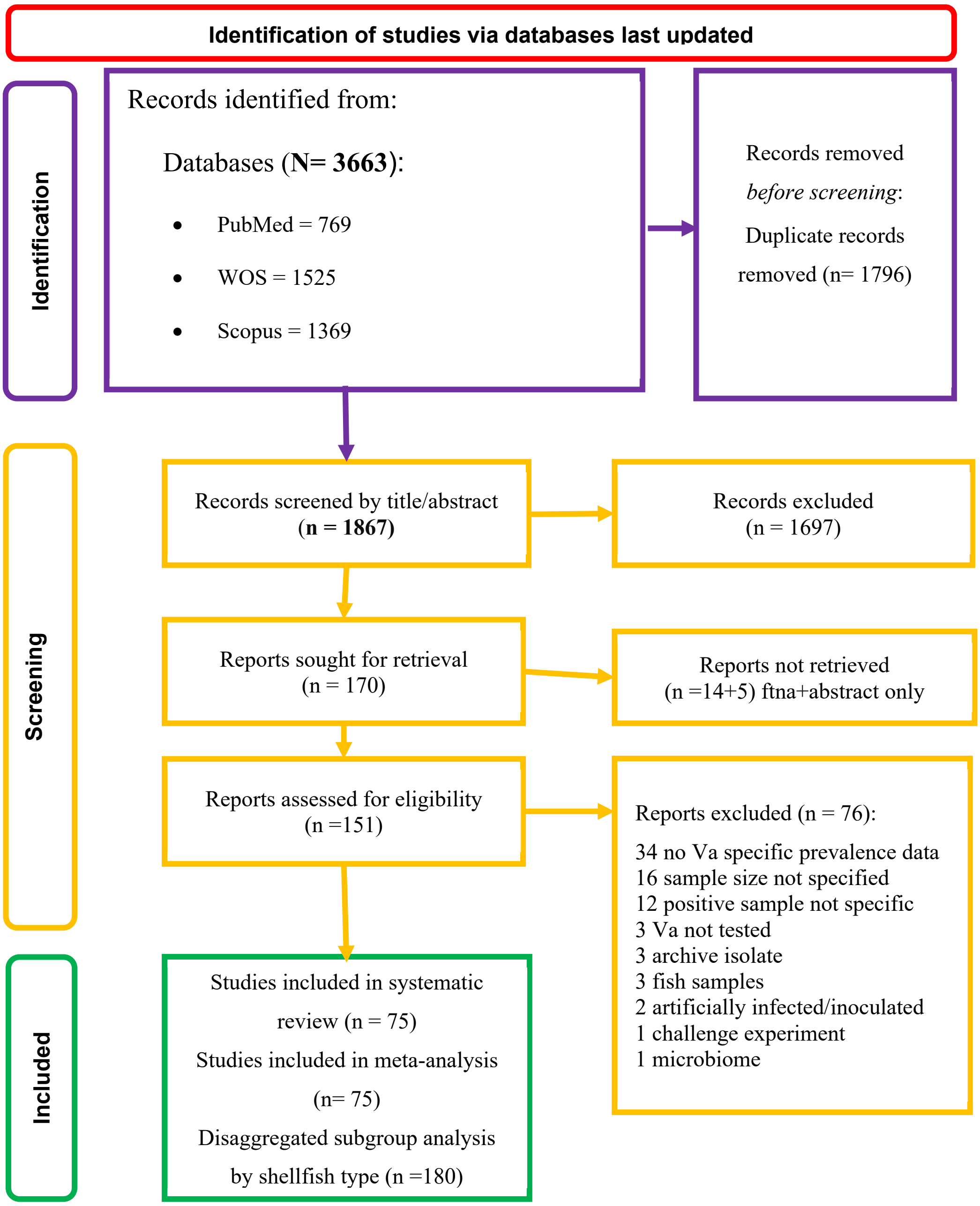

Figure S1. Flow diagram for processing data sources on shellfish-borne *V. alginolyticus*. ftna=full-text not available, Va= *V. alginolyticus*.

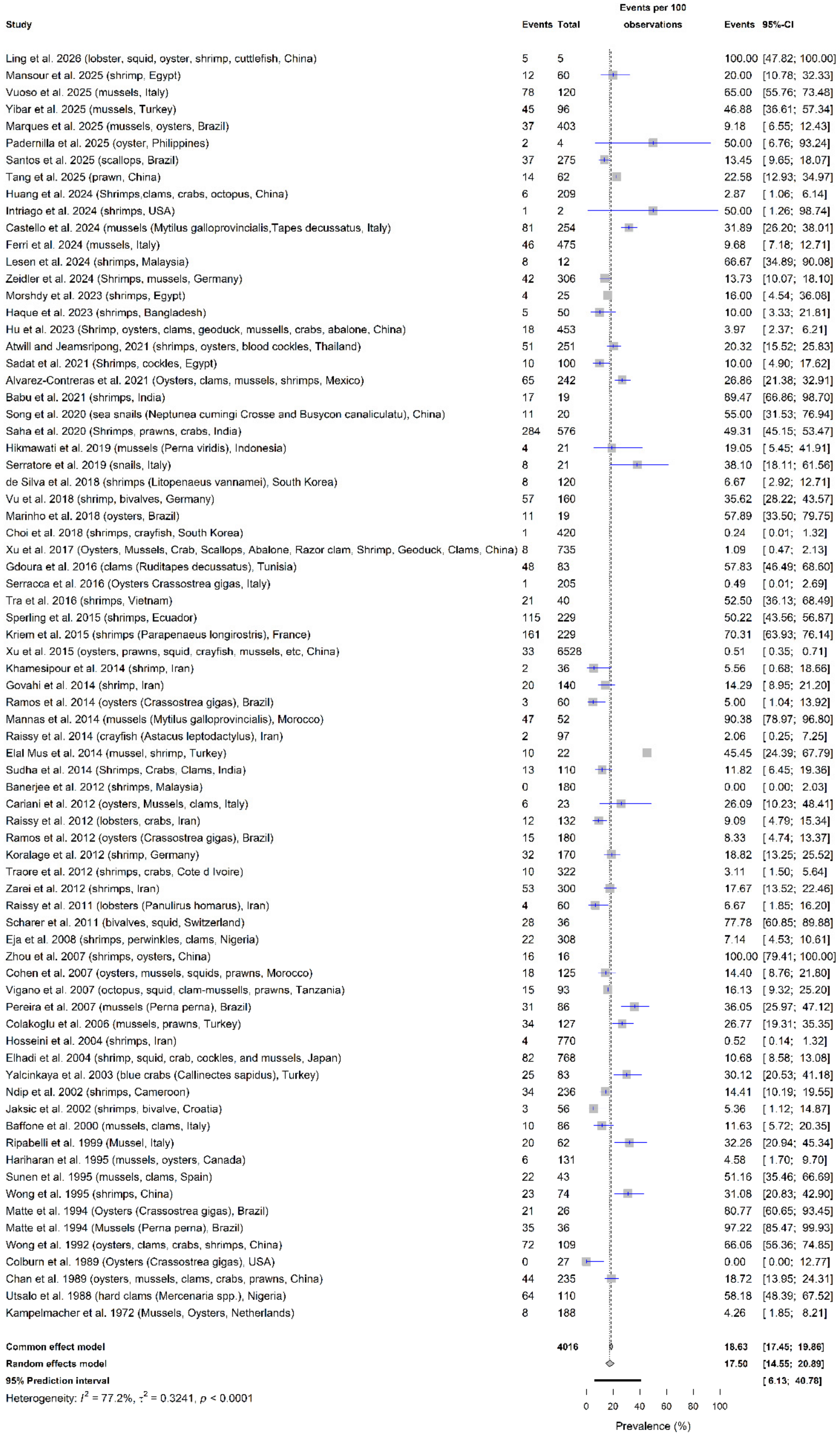

Figure S2a: Sensitivity analysis of prevalence of *V. alginolyticus* contamination in shellfish based on outliers removal.

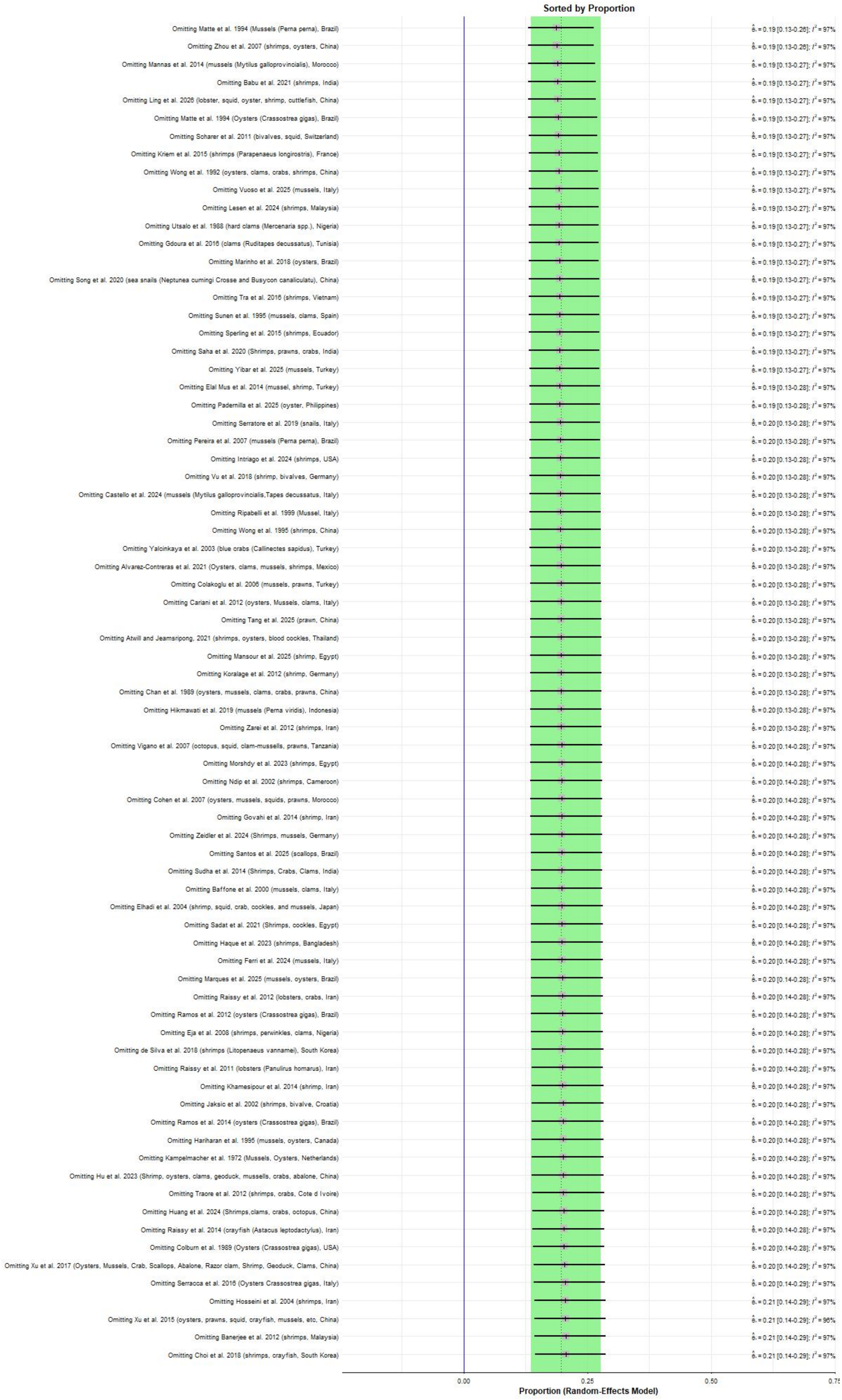

Sorted by Proportion
Omitting Matte et al. 1994 (Mussels (Perna perna), Brazil) — $\hat{\theta}$. = 0.19 [0.13-0.26]; I^2 = 97%
Omitting Zhou et al. 2007 (shrimps, oysters, China) — $\hat{\theta}$. = 0.19 [0.13-0.26]; I^2 = 97%
Omitting Mannas et al. 2014 (mussels (Mytilus galloprovincialis), Morocco) — $\hat{\theta}$. = 0.19 [0.13-0.27]; I^2 = 97%
Omitting Babu et al. 2021 (shrimps, India) — $\hat{\theta}$. = 0.19 [0.13-0.27]; I^2 = 97%
Omitting Ling et al. 2026 (lobster, squid, oyster, shrimp, cuttlefish, China) — $\hat{\theta}$. = 0.19 [0.13-0.27]; I^2 = 97%
Omitting Matte et al. 1994 (Oysters (Crassostrea gigas), Brazil) — $\hat{\theta}$. = 0.19 [0.13-0.27]; I^2 = 97%
Omitting Scharer et al. 2011 (bivalves, squid, Switzerland) — $\hat{\theta}$. = 0.19 [0.13-0.27]; I^2 = 97%
Omitting Kriem et al. 2015 (shrimps (Parapenaeus longirostris), France) — $\hat{\theta}$. = 0.19 [0.13-0.27]; I^2 = 97%
Omitting Wong et al. 1992 (oysters, clams, crabs, shrimps, China) — $\hat{\theta}$. = 0.19 [0.13-0.27]; I^2 = 97%
Omitting Vuoso et al. 2025 (mussels, Italy) — $\hat{\theta}$. = 0.19 [0.13-0.27]; I^2 = 97%
Omitting Lesen et al. 2024 (shrimps, Malaysia) — $\hat{\theta}$. = 0.19 [0.13-0.27]; I^2 = 97%
Omitting Utsalo et al. 1988 (hard clams (Mercenaria spp.), Nigeria) — $\hat{\theta}$. = 0.19 [0.13-0.27]; I^2 = 97%
Omitting Gdoura et al. 2016 (clams (Ruditapes decussatus), Tunisia) — $\hat{\theta}$. = 0.19 [0.13-0.27]; I^2 = 97%
Omitting Marinho et al. 2018 (oysters, Brazil) — $\hat{\theta}$. = 0.19 [0.13-0.27]; I^2 = 97%
Omitting Song et al. 2020 (sea snails (Neptunea cumingi Crosse and Busycon canaliculatu), China) — $\hat{\theta}$. = 0.19 [0.13-0.27]; I^2 = 97%
Omitting Tra et al. 2016 (shrimps, Vietnam) — $\hat{\theta}$. = 0.19 [0.13-0.27]; I^2 = 97%
Omitting Sunen et al. 1995 (mussels, clams, Spain) — $\hat{\theta}$. = 0.19 [0.13-0.27]; I^2 = 97%
Omitting Sperling et al. 2015 (shrimps, Ecuador) — $\hat{\theta}$. = 0.19 [0.13-0.27]; I^2 = 97%
Omitting Saha et al. 2020 (Shrimps, prawns, crabs, India) — $\hat{\theta}$. = 0.19 [0.13-0.27]; I^2 = 97%
Omitting Yibar et al. 2025 (mussels, Turkey) — $\hat{\theta}$. = 0.19 [0.13-0.27]; I^2 = 97%
Omitting Elal Mus et al. 2014 (mussel, shrimp, Turkey) — $\hat{\theta}$. = 0.19 [0.13-0.28]; I^2 = 97%
Omitting Padernilla et al. 2025 (oyster, Philippines) — $\hat{\theta}$. = 0.19 [0.13-0.28]; I^2 = 97%
Omitting Serratore et al. 2019 (snails, Italy) — $\hat{\theta}$. = 0.20 [0.13-0.28]; I^2 = 97%
Omitting Pereira et al. 2007 (mussels (Perna perna), Brazil) — $\hat{\theta}$. = 0.20 [0.13-0.28]; I^2 = 97%
Omitting Intriago et al. 2024 (shrimps, USA) — $\hat{\theta}$. = 0.20 [0.13-0.28]; I^2 = 97%
Omitting Vu et al. 2018 (shrimp, bivalves, Germany) — $\hat{\theta}$. = 0.20 [0.13-0.28]; I^2 = 97%
Omitting Castello et al. 2024 (mussels (Mytilus galloprovincialis,Tapes decussatus, Italy) — $\hat{\theta}$. = 0.20 [0.13-0.28]; I^2 = 97%
Omitting Ripabelli et al. 1999 (Mussel, Italy) — $\hat{\theta}$. = 0.20 [0.13-0.28]; I^2 = 97%
Omitting Wong et al. 1995 (shrimps, China) — $\hat{\theta}$. = 0.20 [0.13-0.28]; I^2 = 97%
Omitting Yalcinkaya et al. 2003 (blue crabs (Callinectes sapidus), Turkey) — $\hat{\theta}$. = 0.20 [0.13-0.28]; I^2 = 97%
Omitting Alvarez-Contreras et al. 2021 (Oysters, clams, mussels, shrimps, Mexico) — $\hat{\theta}$. = 0.20 [0.13-0.28]; I^2 = 97%
Omitting Colakoglu et al. 2006 (mussels, prawns, Turkey) — $\hat{\theta}$. = 0.20 [0.13-0.28]; I^2 = 97%
Omitting Cariani et al. 2012 (oysters, Mussels, clams, Italy) — $\hat{\theta}$. = 0.20 [0.13-0.28]; I^2 = 97%
Omitting Tang et al. 2025 (prawn, China) — $\hat{\theta}$. = 0.20 [0.13-0.28]; I^2 = 97%
Omitting Atwill and Jeamsripong, 2021 (shrimps, oysters, blood cockles, Thailand) — $\hat{\theta}$. = 0.20 [0.13-0.28]; I^2 = 97%
Omitting Mansour et al. 2025 (shrimp, Egypt) — $\hat{\theta}$. = 0.20 [0.13-0.28]; I^2 = 97%
Omitting Koralage et al. 2012 (shrimp, Germany) — $\hat{\theta}$. = 0.20 [0.13-0.28]; I^2 = 97%
Omitting Chan et al. 1989 (oysters, mussels, clams, crabs, prawns, China) — $\hat{\theta}$. = 0.20 [0.13-0.28]; I^2 = 97%
Omitting Hikmawati et al. 2019 (mussels (Perna viridis), Indonesia) — $\hat{\theta}$. = 0.20 [0.13-0.28]; I^2 = 97%
Omitting Zarei et al. 2012 (shrimps, Iran) — $\hat{\theta}$. = 0.20 [0.13-0.28]; I^2 = 97%
Omitting Vigano et al. 2007 (octopus, squid, clam-mussels, prawns, Tanzania) — $\hat{\theta}$. = 0.20 [0.14-0.28]; I^2 = 97%
Omitting Morshdy et al. 2023 (shrimps, Egypt) — $\hat{\theta}$. = 0.20 [0.14-0.28]; I^2 = 97%
Omitting Ndip et al. 2002 (shrimps, Cameroon) — $\hat{\theta}$. = 0.20 [0.14-0.28]; I^2 = 97%
Omitting Cohen et al. 2007 (oysters, mussels, squids, prawns, Morocco) — $\hat{\theta}$. = 0.20 [0.14-0.28]; I^2 = 97%
Omitting Govahi et al. 2014 (shrimp, Iran) — $\hat{\theta}$. = 0.20 [0.14-0.28]; I^2 = 97%
Omitting Zeidler et al. 2024 (Shrimps, mussels, Germany) — $\hat{\theta}$. = 0.20 [0.14-0.28]; I^2 = 97%
Omitting Santos et al. 2025 (scallops, Brazil) — $\hat{\theta}$. = 0.20 [0.14-0.28]; I^2 = 97%
Omitting Sudha et al. 2014 (Shrimps, Crabs, Clams, India) — $\hat{\theta}$. = 0.20 [0.14-0.28]; I^2 = 97%
Omitting Baffone et al. 2000 (mussels, clams, Italy) — $\hat{\theta}$. = 0.20 [0.14-0.28]; I^2 = 97%
Omitting Elhadi et al. 2004 (shrimp, squid, crab, cockles, and mussels, Japan) — $\hat{\theta}$. = 0.20 [0.14-0.28]; I^2 = 97%
Omitting Sadat et al. 2021 (Shrimps, cockles, Egypt) — $\hat{\theta}$. = 0.20 [0.14-0.28]; I^2 = 97%
Omitting Haque et al. 2023 (shrimps, Bangladesh) — $\hat{\theta}$. = 0.20 [0.14-0.28]; I^2 = 97%
Omitting Ferri et al. 2024 (mussels, Italy) — $\hat{\theta}$. = 0.20 [0.14-0.28]; I^2 = 97%
Omitting Marques et al. 2025 (mussels, oysters, Brazil) — $\hat{\theta}$. = 0.20 [0.14-0.28]; I^2 = 97%
Omitting Raissy et al. 2012 (lobsters, crabs, Iran) — $\hat{\theta}$. = 0.20 [0.14-0.28]; I^2 = 97%
Omitting Ramos et al. 2012 (oysters (Crassostrea gigas), Brazil) — $\hat{\theta}$. = 0.20 [0.14-0.28]; I^2 = 97%
Omitting Eja et al. 2008 (shrimps, periwinkles, clams, Nigeria) — $\hat{\theta}$. = 0.20 [0.14-0.28]; I^2 = 97%
Omitting de Silva et al. 2018 (shrimps (Litopenaeus vannamei), South Korea) — $\hat{\theta}$. = 0.20 [0.14-0.28]; I^2 = 97%
Omitting Raissy et al. 2011 (lobsters (Panulirus homarus), Iran) — $\hat{\theta}$. = 0.20 [0.14-0.28]; I^2 = 97%
Omitting Khamesipour et al. 2014 (shrimp, Iran) — $\hat{\theta}$. = 0.20 [0.14-0.28]; I^2 = 97%
Omitting Jaksic et al. 2002 (shrimps, bivalve, Croatia) — $\hat{\theta}$. = 0.20 [0.14-0.28]; I^2 = 97%
Omitting Ramos et al. 2014 (oysters (Crassostrea gigas), Brazil) — $\hat{\theta}$. = 0.20 [0.14-0.28]; I^2 = 97%
Omitting Hariharan et al. 1995 (mussels, oysters, Canada) — $\hat{\theta}$. = 0.20 [0.14-0.28]; I^2 = 97%
Omitting Kampelmacher et al. 1972 (Mussels, Oysters, Netherlands) — $\hat{\theta}$. = 0.20 [0.14-0.28]; I^2 = 97%
Omitting Hu et al. 2023 (Shrimp, oysters, clams, geoduck, mussells, crabs, abalone, China) — $\hat{\theta}$. = 0.20 [0.14-0.28]; I^2 = 97%
Omitting Traore et al. 2012 (shrimps, crabs, Cote d Ivoire) — $\hat{\theta}$. = 0.20 [0.14-0.28]; I^2 = 97%
Omitting Huang et al. 2024 (Shrimps,clams, crabs, octopus, China) — $\hat{\theta}$. = 0.20 [0.14-0.28]; I^2 = 97%
Omitting Raissy et al. 2014 (crayfish (Astacus leptodactylus), Iran) — $\hat{\theta}$. = 0.20 [0.14-0.28]; I^2 = 97%
Omitting Colburn et al. 1989 (Oysters (Crassostrea gigas), USA) — $\hat{\theta}$. = 0.20 [0.14-0.28]; I^2 = 97%
Omitting Xu et al. 2017 (Oysters, Mussels, Crab, Scallops, Abalone, Razor clam, Shrimp, Geoduck, Clams, China) — $\hat{\theta}$. = 0.20 [0.14-0.29]; I^2 = 97%
Omitting Serracca et al. 2016 (Oysters Crassostrea gigas, Italy) — $\hat{\theta}$. = 0.20 [0.14-0.29]; I^2 = 97%
Omitting Hosseini et al. 2004 (shrimps, Iran) — $\hat{\theta}$. = 0.21 [0.14-0.29]; I^2 = 97%
Omitting Xu et al. 2015 (oysters, prawns, squid, crayfish, mussels, etc, China) — $\hat{\theta}$. = 0.21 [0.14-0.29]; I^2 = 96%
Omitting Banerjee et al. 2012 (shrimps, Malaysia) — $\hat{\theta}$. = 0.21 [0.14-0.29]; I^2 = 97%
Omitting Choi et al. 2018 (shrimps, crayfish, South Korea) — $\hat{\theta}$. = 0.21 [0.14-0.29]; I^2 = 97%
0.00
0.25
0.50
0.75
Proportion (Random-Effects Model)

Figure S2b: Leave-out-sensitivity analysis outcomes of *V. alginolyticus* contamination prevalence in shellfish.

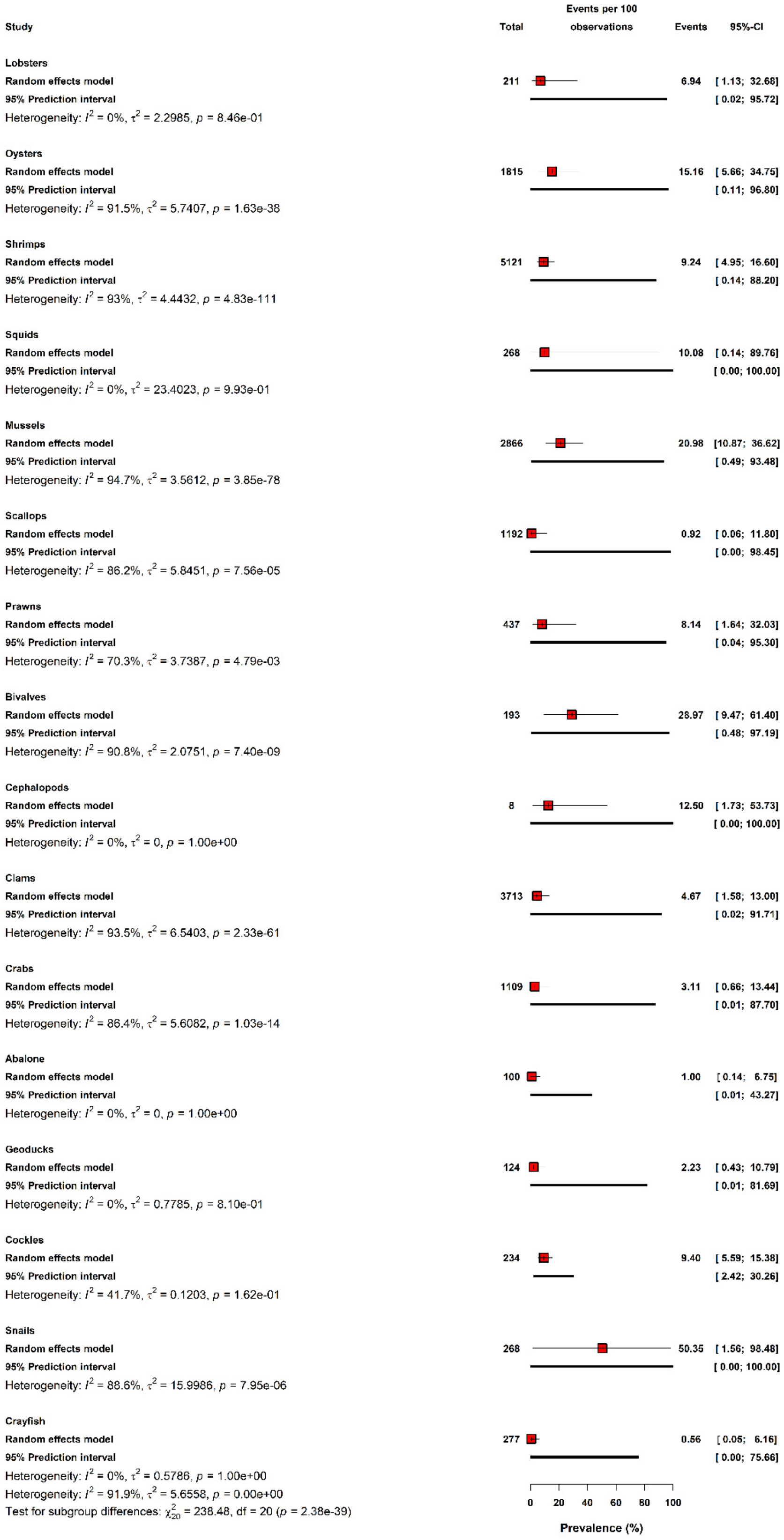

Figure S3a: Prevalence of *V. alginolyticus* in different shellfish types.

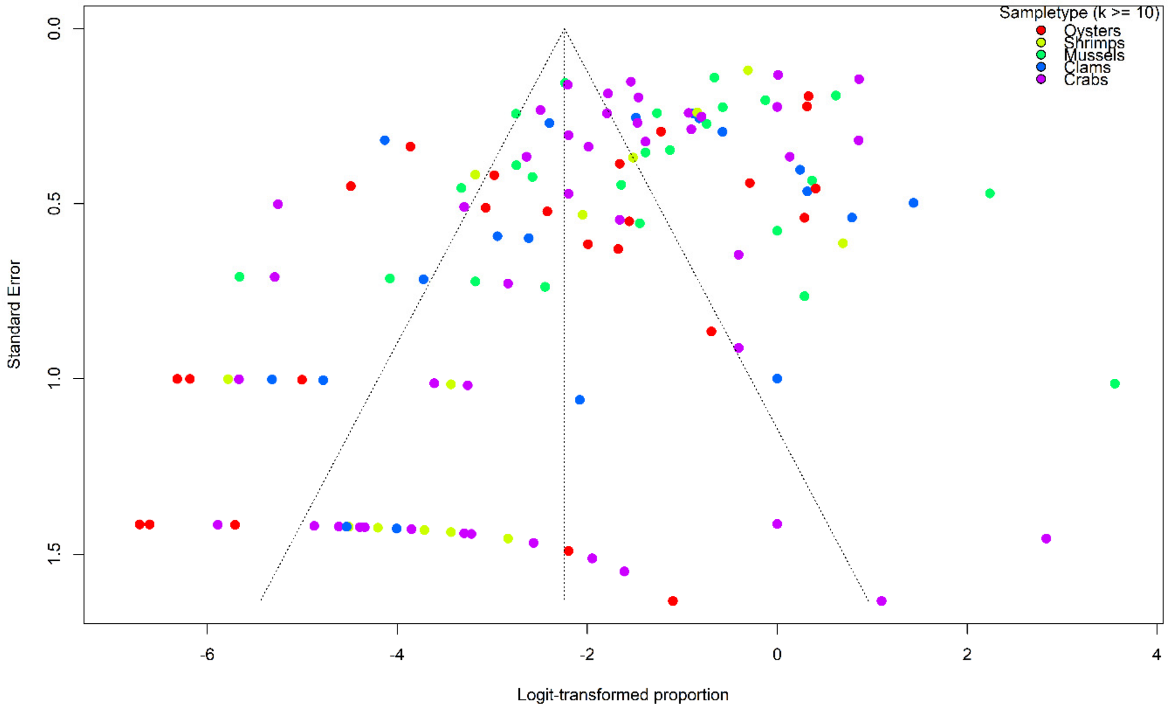

Figure S3b: Subgroup funnel plot for *V. alginolyticus* prevalence in different shellfish types. Oysters (β_0 = -0.578, p = 0.71), shrimps (β_0 = -2.185, p = 0.015), mussels (β_0 = -1.312, p = 0.48), clams (β_0 = -4.729, p = 0.002), and crabs (β_0 = -3.119, p = 0.001).

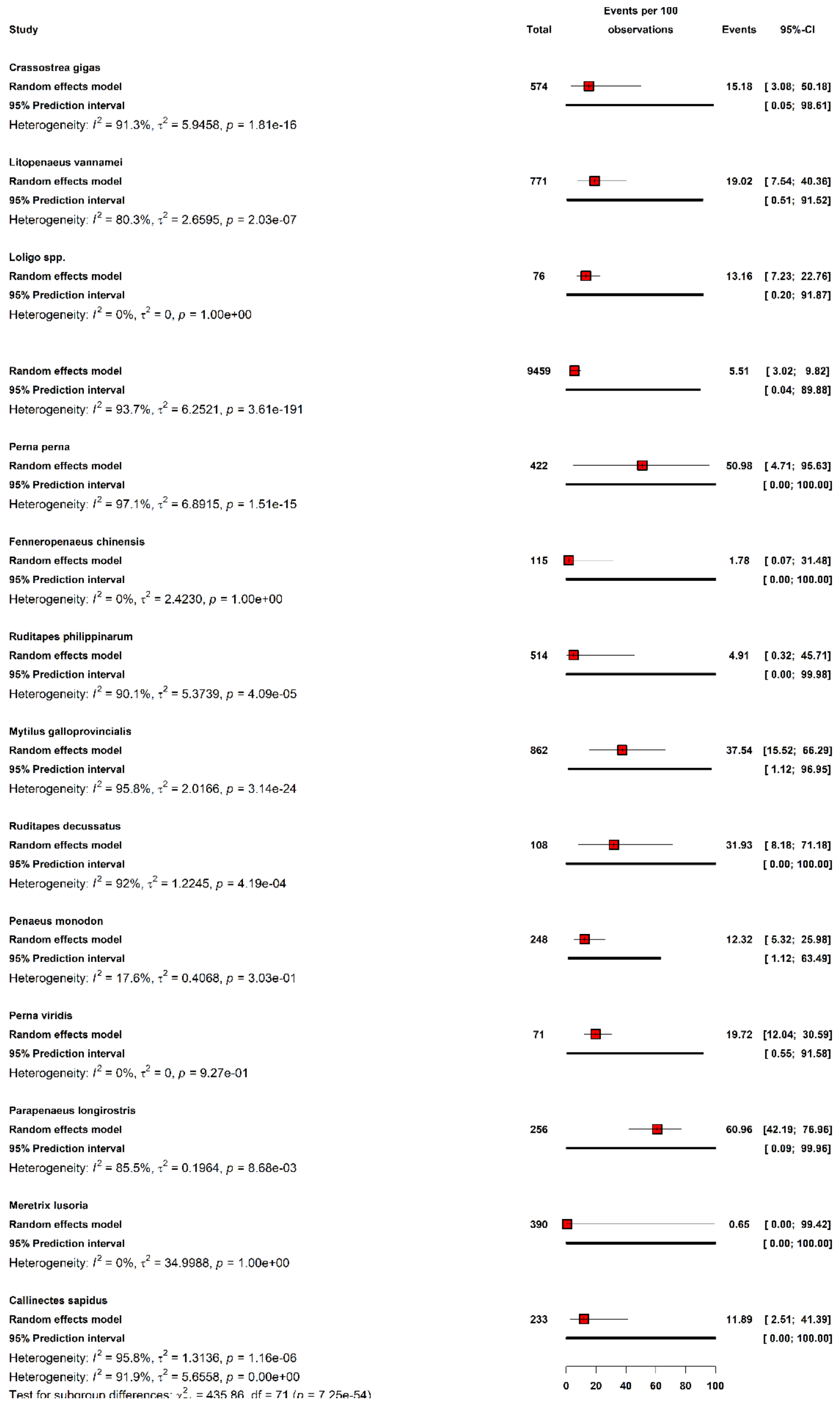

Figure S4: Prevalence of *V. alginolyticus* in different shellfish species.

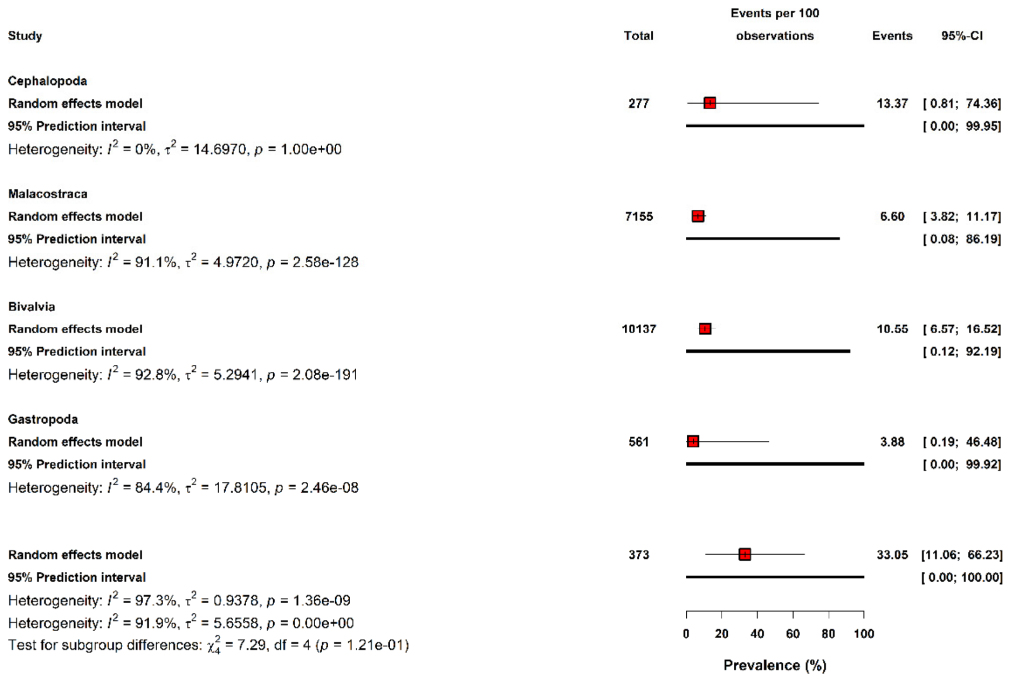

Figure S5a: Prevalence of *V. alginolyticus* in different shellfish classes.

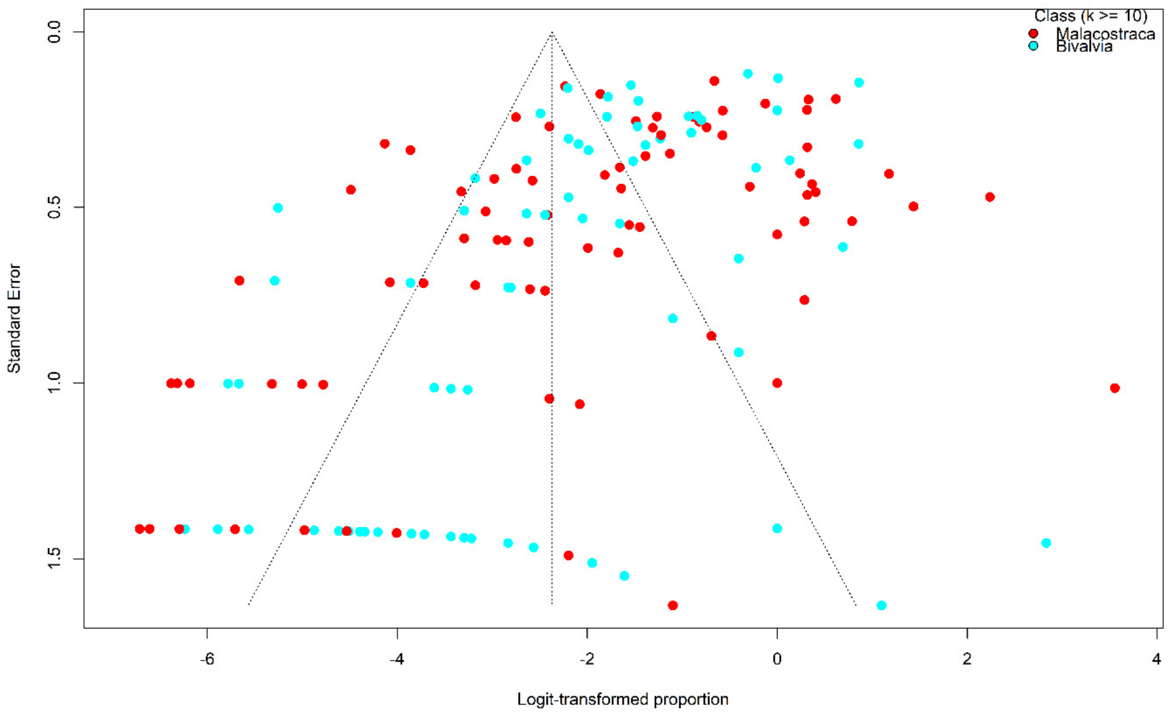

Figure S5b: Subgroup funnel plot for prevalence of *V. alginolyticus* in different shellfish.
classes *Malacostraca* (-2.527, p = 3.29e-05) and *Bivalvia* (-2.355, p = 0.002).

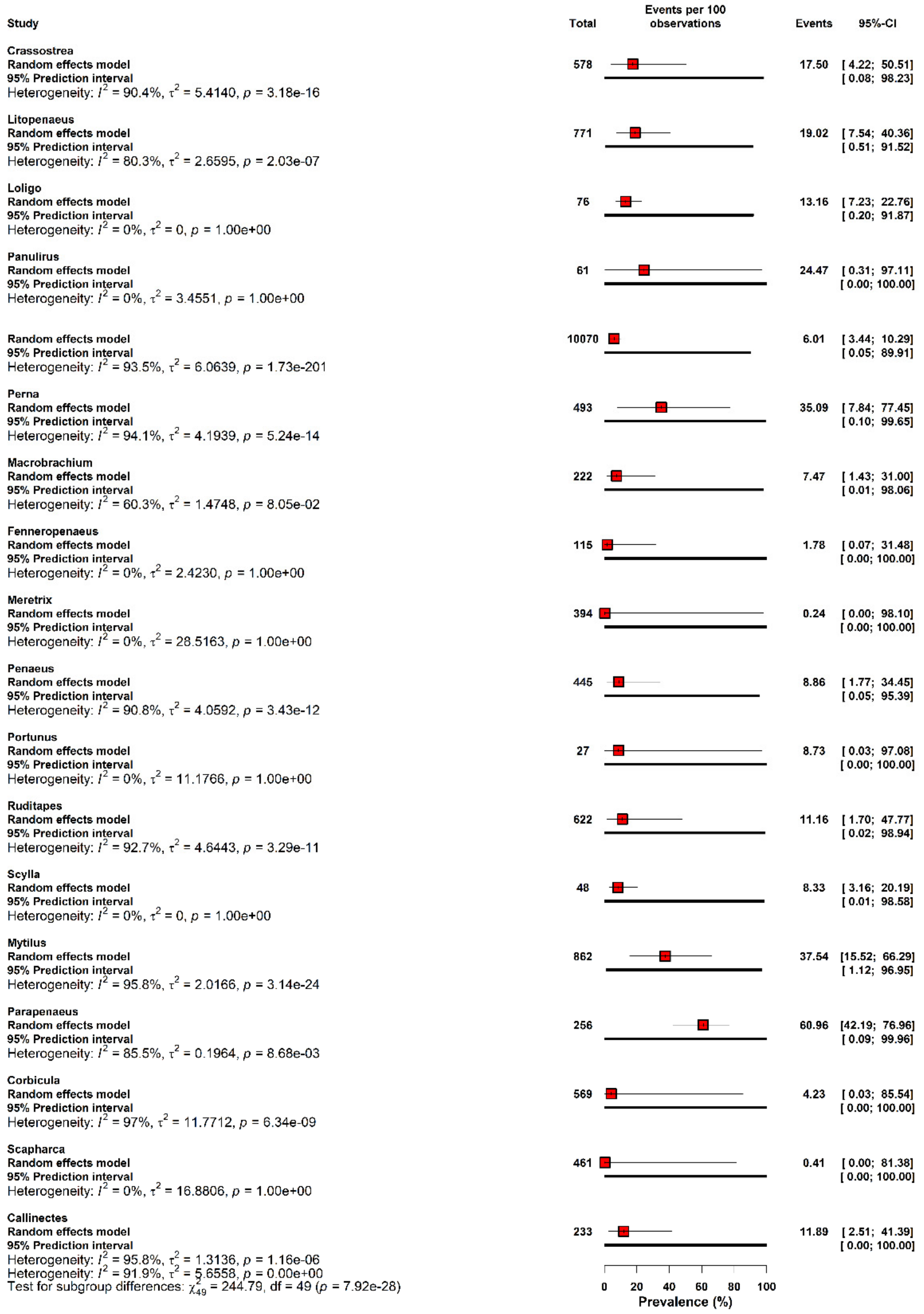

Figure S6a: Prevalence of *V. alginolyticus* in different shellfish genera.

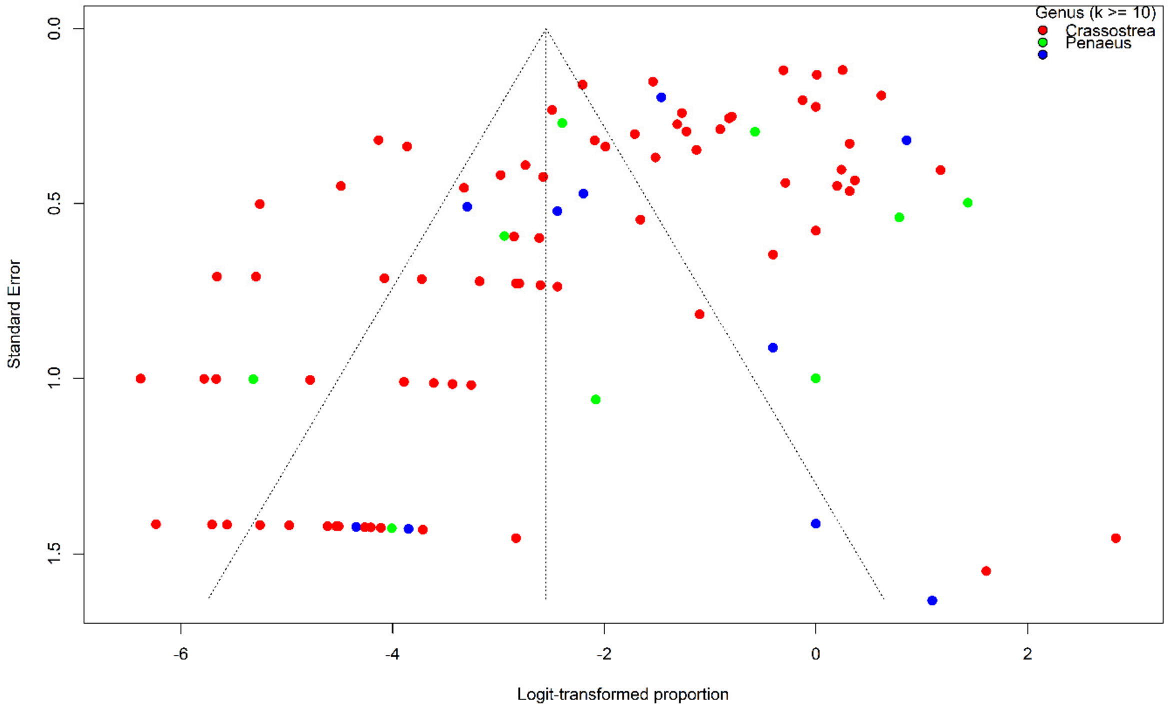

Figure S6b: Subgroup funnel plot for prevalence of *V. alginolyticus* in different shellfish genera: Crassostrea (β_0 = -0.061, p = 0.98); Penaeus (β_0 = -0.824, p = 0.63)

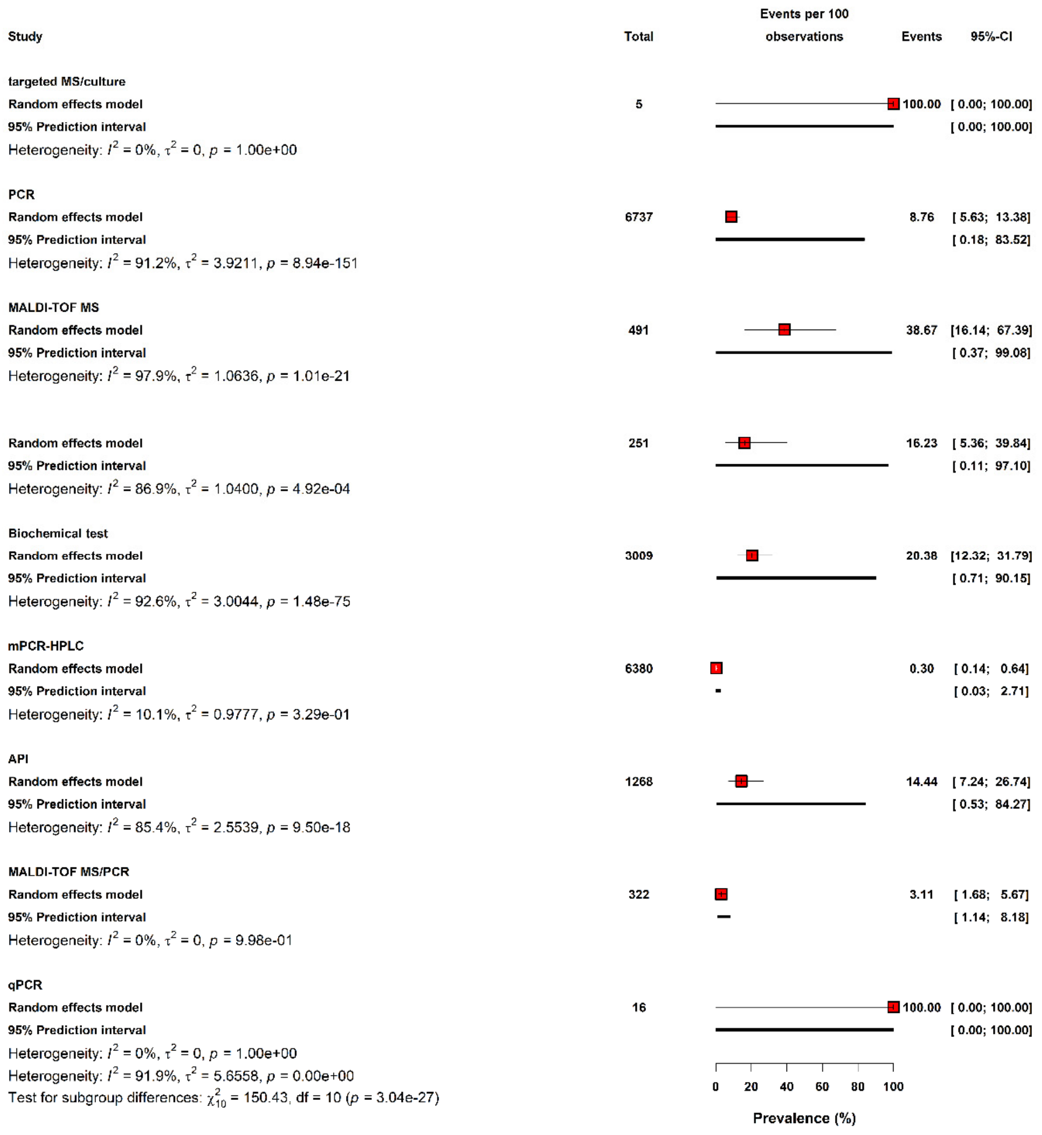

Figure S7a: Prevalence of *V. alginolyticus* in different shellfish by detection assays.

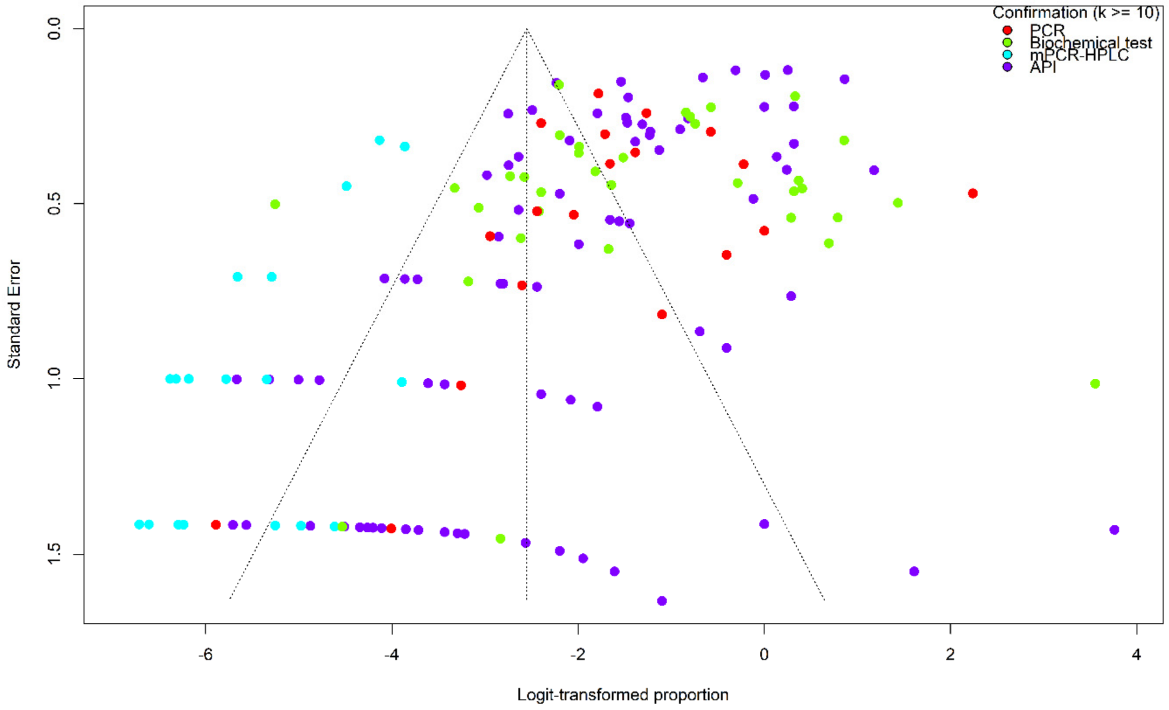

Figure S7b: Subgroup funnel plot for prevalence of *V. alginolyticus* in shellfish by detection assays. API (β_0 = -0.389, p = 0.78), biochemical test (β_0 = -0.754, p = 0.62), PCR (β_0 = -2.541, p = 3.61e-06), and mPCR-HPLC (β_0 = -1.759, p = 6.76e-05).

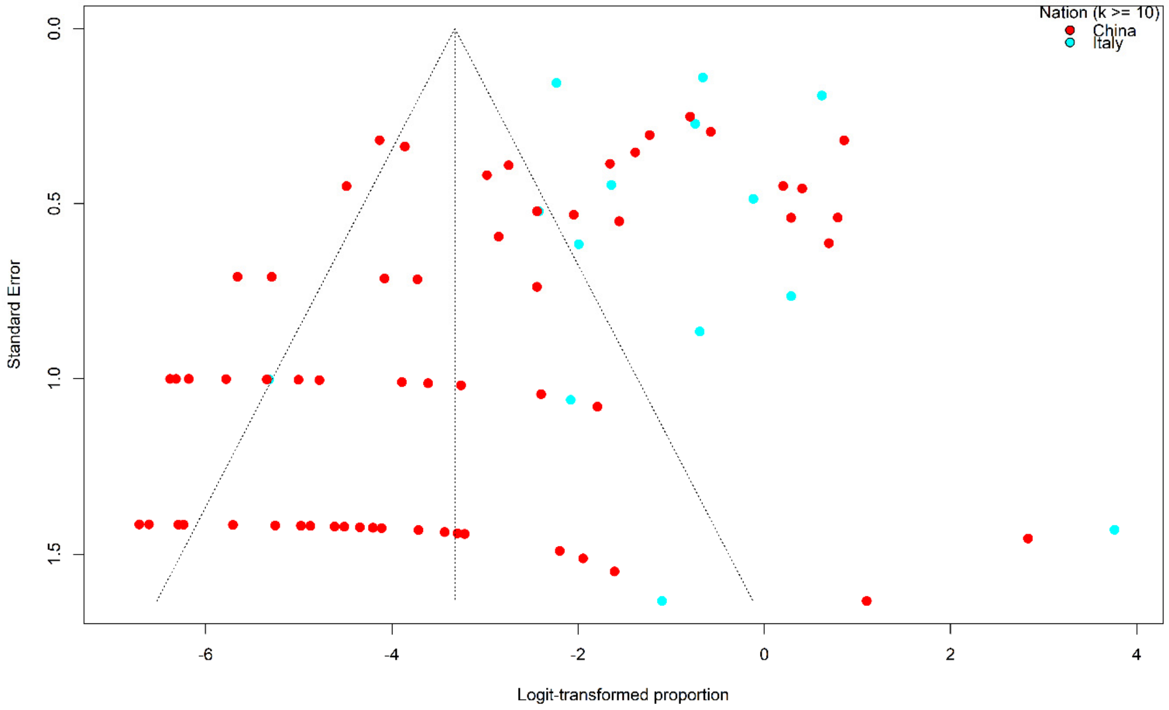

Figure S8. Subgroup funnel plot for prevalence of *V. alginolyticus* by nation. China (β_0 = -1.996, p = 0.003) and Italy (β_0 = -0.273, p = 0.87)

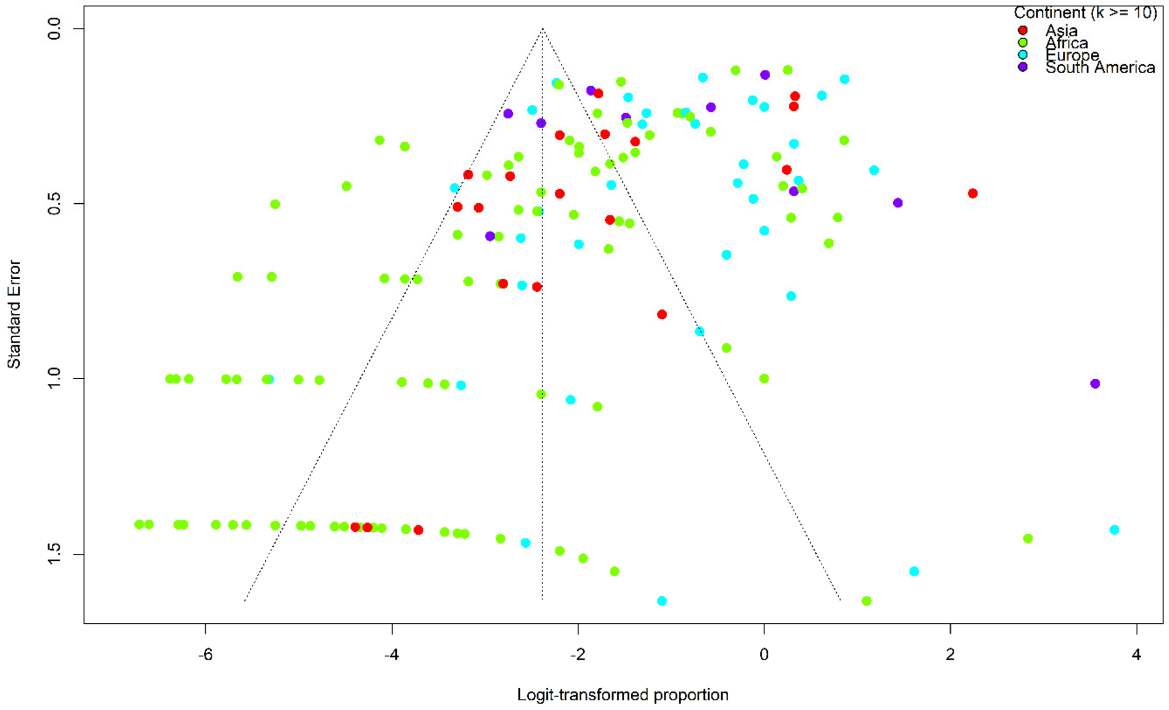

Figure S9: Subgroup funnel plot for prevalence of *V. alginolyticus* in shellfish by continent: Asia (β_0 = -2.657, p = 5.40e-08), Africa (β_0 = -3.287, p = 0.09), Europe (β_0 = -0.997, p = 0.41), and South America (β_0 = -0.032, p = 0.99).

Table S1: Disaggregated dataset for subgroup analysis.

sn	study Index	Author/subgroup identifier	N	P	Type	Species	Genus	Class	Confirmation	Medium	Nation	Continent
1	[4]	Ling et al. 2026 (Cuttlefish, Sepia pharaonis, China)	1	1	Cuttlefish	*Sepia pharaonis*	*Sepia*	*Cephalopoda*	targeted MS/culture		China	Asia
2	[4]	Ling et al. 2026 (Lobsters, Panulirus spp., China)	1	1	Lobsters	*Panulirus* spp.	*Panulirus*	*Malacostraca*	targeted MS/culture		China	Asia
3	[4]	Ling et al. 2026 (Oysters, Crassostrea gigas, China)	1	1	Oysters	*Crassostrea gigas*	*Crassostrea*	*Bivalvia*	targeted MS/culture		China	Asia
4	[4]	Ling et al. 2026 (Shrimps, Penaeus vannamei, China)	1	1	Shrimps	*Litopenaeus vannamei*	*Penaeus*	*Malacostraca*	targeted MS/culture		China	Asia
5	[4]	Ling et al. 2026 (Squids, Loligo spp., China)	1	1	Squids	*Loligo* spp.	*Loligo*	*Cephalopoda*	targeted MS/culture		China	Asia
6	[5]	Mansour et al. 2025 (Shrimps, Litopenaeus vannamei, Egypt)	60	12	Shrimps	*Litopenaeus vannamei*	*Litopenaeus*	*Malacostraca*	PCR	TCBS	Egypt	Africa
7	[6]	Vuoso et al. 2025 (Mussels, Italy)	120	78	Mussels	NRT	NRT	*Bivalvia*	MALDI-TOF MS	ISO 21872-1:2017 method	Italy	Europe
8	[7]	Yibar et al. 2025 (Mussels, Turkey)	96	45	Mussels	NRT	NRT	*Bivalvia*	MALDI-TOF MS	TCBS	Turkey	Europe
9	[8]	Marques et al. 2025 (Mussels, Perna perna, Brazil)	300	18	Mussels	*Perna perna*	*Perna*	*Bivalvia*	PCR	TCBS	Brazil	South America
10	[8]	Marques et al. 2025 (Oysters, Ostreidae, Brazil)	103	19	Oysters	*Ostreidae*	*Ostreidae*	*Bivalvia*	PCR	TCBS	Brazil	South America
11	[9]	Padernilla et al. 2025 (Oysters, Magallana bilineata, Philippines)	4	2	Oysters	*Magallana bilineata*	*Crassostrea*	*Bivalvia*	Sequencing	TCBS	Philippines	Asia
12	[10]	Santos et al. 2025 (Scallops, Nodipecten nodosus, Brazil)	275	37	Scallops	*Nodipecten nodosus*	*Nodipecten*	*Bivalvia*	MALDI-TOF MS	BHI	Brazil	South America
13	[11]	Tang et al. 2025 (Prawns, Macrobrachium rosenbergii, China)	62	14	Prawns	*Macrobrachium rosenbergii*	*Macrobrachium*	*Malacostraca*	PCR	TCBS	China	Asia
14	[32]	Huang et al. 2024 (Bivalves, Solen grandis, China)	12	1	Bivalves	*Solen grandis*	*Solen*	*Bivalvia*	PCR	TCBS	China	Asia
15	[32]	Huang et al. 2024 (Cephalopods, Octopus variabilis, China)	1	0	Cephalopods	*Octopus variabilis*	*Octopus*	*Cephalopoda*	PCR	TCBS	China	Asia
16	[32]	Huang et al. 2024 (Cephalopods, Sepiella maindroni, China)	7	1	Cephalopods	*Sepiella maindroni*	*Sepiella*	*Cephalopoda*	PCR	TCBS	China	Asia
17	[32]	Huang et al. 2024 (Clams, Meretrix meretrix, China)	4	0	Clams	*Meretrix meretrix*	*Meretrix*	*Bivalvia*	PCR	TCBS	China	Asia
18	[32]	Huang et al. 2024 (Clams, Ruditapes philippinarum, China)	23	4	Clams	*Ruditapes philippinarum*	*Ruditapes*	*Bivalvia*	PCR	TCBS	China	Asia
19	[32]	Huang et al. 2024 (Crabs, Portunus trituberculatus, China)	15	0	Crabs	*Portunus trituberculatus*	*Portunus*	*Malacostraca*	PCR	TCBS	China	Asia
20	[32]	Huang et al. 2024 (Crabs, Scylla paramamosain, China)	13	0	Crabs	*Scylla paramamosain*	*Scylla*	*Malacostraca*	PCR	TCBS	China	Asia
21	[32]	Huang et al. 2024 (Shrimps, Exopalaemon carinicauda, China)	3	0	Shrimps	*Exopalaemon carinicauda*	*Exopalaemon*	*Malacostraca*	PCR	TCBS	China	Asia
22	[32]	Huang et al. 2024 (Shrimps, Fenneropenaeus chinensis, China)	65	0	Shrimps	*Fenneropenaeus chinensis*	*Fenneropenaeus*	*Malacostraca*	PCR	TCBS	China	Asia
23	[32]	Huang et al. 2024 (Shrimps, Oratosquilla oratoria, China)	1	0	Shrimps	*Oratosquilla oratoria*	*Oratosquilla*	*Malacostraca*	PCR	TCBS	China	Asia
24	[32]	Huang et al. 2024 (Shrimps, Palaemon gravieri, China)	13	0	Shrimps	*Palaemon gravieri*	*Palaemon*	*Malacostraca*	PCR	TCBS	China	Asia
25	[32]	Huang et al. 2024 (Shrimps, Parapenaeopsis hardwickii, China)	12	0	Shrimps	*Parapenaeopsis hardwickii*	*Parapenaeopsis*	*Malacostraca*	PCR	TCBS	China	Asia
26	[32]	Huang et al. 2024 (Shrimps, Penaeus japonicus, China)	38	0	Shrimps	*Penaeus japonicus*	*Penaeus*	*Malacostraca*	PCR	TCBS	China	Asia
27	[32]	Huang et al. 2024 (Shrimps, Trachypenaeus curvirostris, China)	2	0	Shrimps	*Trachypenaeus curvirostris*	*Trachypenaeus*	*Malacostraca*	PCR	TCBS	China	Asia
28	[33]	Intriago et al. 2024 (Shrimps, Penaeus vannamei, USA)	2	1	Shrimps	*Litopenaeus vannamei*	*Penaeus*	*Malacostraca*	PCR	TCBS/CHROMagar	USA	North America

29	[34]	Castello et al. 2024 (Clams, Ruditapes decussatus, Italy)	25	3	Clams	*Ruditapes decussatus*	*Ruditapes*	*Bivalvia*	PCR	TCBS/CHROMagar	Italy	Europe
30	[34]	Castello et al. 2024 (Mussels, Mytilus galloprovincialis, Italy)	229	78	Mussels	*Mytilus galloprovincialis*	*Mytilus*	*Bivalvia*	PCR	TCBS/CHROMagar	Italy	Europe
31	[35]	Ferri et al. 2024 (Mussels, Mytilus galloprovincialis, Italy)	475	46	Mussels	*Mytilus galloprovincialis*	*Mytilus*	*Bivalvia*	PCR	TCBS	Italy	Europe
32	[36]	Lesen et al. 2024 (Shrimps, Litopenaeus vannamei, Malaysia)	12	8	Shrimps	*Litopenaeus vannamei*	*Litopenaeus*	*Malacostraca*	PCR	APW	Malaysia	Asia
33	[17]	Zeidler et al. 2024 (Bivalves, Mytilus edulis, Ostreidae spp., Pecten jacobaeus, Germany)	38	22	Bivalves	*Mytilus edulis, Ostreidae spp., Pecten jacobaeus*	NRT	*Bivalvia*	PCR	TCBS/Vibrio ChromoSelect Agar (VCS	Germany	Europe
34	[17]	Zeidler et al. 2024 (Shrimps, Penaeus monodon, Litopenaeus vannamei, Germany)	262	20	Shrimps	*Penaeus monodon, Litopenaeus vannamei*	NRT	*Malacostraca*	PCR	TCBS/Vibrio ChromoSelect Agar (VCS	Germany	Europe
35	[17]	Zeidler et al. 2024 (Shrimps, Pleoticus muelleri, Germany)	6	0	Shrimps	*Pleoticus muelleri*	*Pleoticus*	*Malacostraca*	PCR	TCBS/Vibrio ChromoSelect Agar (VCS	Germany	Europe
36	[37]	Morshdy et al. 2023 (Shrimps, Egypt)	25	4	Shrimps	NRT	NRT	*Malacostraca*	PCR	TCBS	Egypt	Africa
37	[38]	Haque et al. 2023 (Shrimps, Penaeus monodon, Bangladesh)	50	5	Shrimps	*Penaeus monodon*	*Penaeus*	*Malacostraca*	PCR	TCBS	Bangladesh	Asia
38	[39]	Hu et al. 2023 (Abalone, China)	20	0	Abalone	NRT	NRT	*Gastropoda*	PCR	APW	China	Asia
39	[39]	Hu et al. 2023 (Clams, China)	124	6	Clams	NRT	NRT	*Bivalvia*	PCR	APW	China	Asia
40	[39]	Hu et al. 2023 (Crabs, China)	45	0	Crabs	NRT	NRT	*Malacostraca*	PCR	APW	China	Asia
41	[39]	Hu et al. 2023 (Geoducks, China)	25	2	Geoducks	NRT	NRT	*Bivalvia*	PCR	APW	China	Asia
42	[39]	Hu et al. 2023 (Mussels, China)	116	7	Mussels	NRT	NRT	*Bivalvia*	PCR	APW	China	Asia
43	[39]	Hu et al. 2023 (Oysters, China)	85	2	Oysters	NRT	NRT	*Bivalvia*	PCR	APW	China	Asia
44	[39]	Hu et al. 2023 (Shrimps, China)	38	1	Shrimps	NRT	NRT	*Malacostraca*	PCR	APW	China	Asia
45	[40]	Atwill and Jeamsripong, 2021 (Cockles, Tegillarca granosa, Thailand)	84	3	Cockles	*Tegillarca granosa*	*Tegillarca*	*Bivalvia*		TCBS/CHROMagar	Thailand	Asia
46	[40]	Atwill and Jeamsripong, 2021 (Oysters, Saccostrea cucculata, Thailand)	82	24	Oysters	*Saccostrea cucculata*	*Saccostrea*	*Bivalvia*		TCBS/CHROMagar	Thailand	Asia
47	[40]	Atwill and Jeamsripong, 2021 (Shrimps, Litopenaeus vannamei, Thailand)	85	24	Shrimps	*Litopenaeus vannamei*	*Litopenaeus*	*Malacostraca*		TCBS/CHROMagar	Thailand	Asia
48	[41]	Sadat et al. 2021 (Cockles, Cerastoderma edule, Egypt)	50	5	Cockles	*Cerastoderma edule*	*Cerastoderma*	*Bivalvia*	PCR	TCBS	Egypt	Africa
49	[41]	Sadat et al. 2021 (Shrimps, Litopenaeus vannamei, Egypt)	50	5	Shrimps	*Litopenaeus vannamei*	*Litopenaeus*	*Malacostraca*	PCR	TCBS	Egypt	Africa
50	[21]	Alvarez-Contreras et al. 2021 (Clams, Mexico)	66	15	Clams	NRT	NRT	*Bivalvia*	PCR	TCBS	Mexico	North America
51	[21]	Alvarez-Contreras et al. 2021 (Mussels, Mexico)	45	11	Mussels	NRT	NRT	*Bivalvia*	PCR	TCBS	Mexico	North America
52	[21]	Alvarez-Contreras et al. 2021 (Oysters, Mexico)	72	22	Oysters	NRT	NRT	*Bivalvia*	PCR	TCBS	Mexico	North America
53	[21]	Alvarez-Contreras et al. 2021 (Shrimps, Mexico)	59	17	Shrimps	NRT	NRT	*Malacostraca*	PCR	TCBS	Mexico	North America
54	[42]	Babu et al. 2021 (Shrimps, Litopenaeus vannamei, India)	91	17	Shrimps	*Litopenaeus vannamei*	*Litopenaeus*	*Malacostraca*	PCR	TCBS	India	Asia
55	[42]	Babu et al. 2021 (Shrimps, Penaeus monodon, India)	23	0	Shrimps	*Penaeus monodon*	*Penaeus*	*Malacostraca*	PCR	TCBS	India	Asia
56	[43	Song et al. 2020 (Snails, Neptunea cumingi Crosse, Busycon canaliculatu, China)	20	11	Snails	*Neptunea cumingi Crosse, Busycon canaliculatu*	NRT	*Gastropoda*	VITEK	TCBS	China	Asia
57	[44]	Saha et al. 2020 (Crabs, India)	288	122	Crabs	NRT	NRT	*Malacostraca*	PCR	TCBS	India	Asia
58	[44]	Saha et al. 2020 (shrimp, prawns, India)	288	162	shrimp, prawns	NRT	NRT	*NRT*	PCR	TCBS	India	Asia
59	[45]	Hikmawati et al. 2019 (Mussels, Perna viridis, Indonesia)	21	4	Mussels	*Perna viridis*	Perna	*Bivalvia*	PCR	TCBS	Indonesia	Asia

60	[46]	Serratore et al. 2019 (Snails, Bolinus brandaris, Italy)	17	8	Snails	*Bolinus brandaris*	Bolinus	*Gastropoda*	PCR	TCBS/CHROMagar Vibrio	Italy	Europe
61	[46]	Serratore et al. 2019 (Snails, Nassarius mutabilis, Italy)	21	21	Snails	*Nassarius mutabilis*	Nassarius	*Gastropoda*	PCR	TCBS/CHROMagar Vibrio	Italy	Europe
62	[47]	de Silva et al. 2018 (Shrimps, Litopenaeus vannamei, South Korea)	120	8	Shrimps	*Litopenaeus vannamei*	Litopenaeus	*Malacostraca*	PCR	TCBS	South Korea	Asia
63	[48]	Vu et al. 2018 (Bivalves, Germany)	80	17	Bivalves	NRT	NRT	*Bivalvia*	PCR	ISO/TS 21872	Germany	Europe
64	[48]	Vu et al. 2018 (Shrimps, Germany)	80	40	Shrimps	NRT	NRT	*Malacostraca*	PCR	ISO/TS 21872	Germany	Europe
65	[49]	Marinho et al. 2018 (Oysters, Brazil)	19	11	Oysters	NRT	NRT	*Bivalvia*	Biochemical test	TCBS	Brazil	South America
66	[50]	Choi et al. 2018 (Crayfish, South Korea)	130	0	Crayfish	NRT	NRT	*Malacostraca*	PCR	TCBS	South Korea	Asia
67	[50]	Choi et al. 2018 (Shrimps, South Korea)	290	1	Shrimps	NRT	NRT	*Malacostraca*	PCR	TCBS	South Korea	Asia
68	[51]	Xu et al. 2017 (Abalone, China)	30	0	Abalone	NRT	NRT	*Gastropoda*	PCR		China	Asia
69	[51]	Xu et al. 2017 (Clams, Ensis leei , China)	150	1	Clams	*Ensis leei*	Ensis	*Bivalvia*	PCR		China	Asia
70	[51]	Xu et al. 2017 (Clams, China)	150	0	Clams	NRT	NRT	*Bivalvia*	PCR		China	Asia
71	[51]	Xu et al. 2017 (Crabs, China)	33	0	Crabs	NRT	NRT	*Malacostraca*	PCR		China	Asia
72	[51]	Xu et al. 2017 (Geoducks, China)	27	1	Geoducks	NRT	NRT	*Bivalvia*	PCR		China	Asia
73	[51]	Xu et al. 2017 (Mussels, China)	120	2	Mussels	NRT	NRT	*Bivalvia*	PCR		China	Asia
74	[51]	Xu et al. 2017 (Oysters, China)	120	1	Oysters	NRT	NRT	*Bivalvia*	PCR		China	Asia
75	[51]	Xu et al. 2017 (Scallops, China)	55	3	Scallops	NRT	NRT	*Bivalvia*	PCR		China	Asia
76	[51]	Xu et al. 2017 (Shrimps, China)	50	0	Shrimps	NRT	NRT	*Malacostraca*	PCR		China	Asia
77	[52]	Gdoura et al. 2016 (Clams, Ruditapes decussatus, Tunisia)	83	48	Clams	*Ruditapes decussatus*	*Ruditapes*	*Bivalvia*	PCR	ISO/TS 21872-1/TCBS	Tunisia	Africa
78	[53]	Serracca et al. 2016 (Oysters, Crassostrea gigas, Italy)	205	1	Oysters	*Crassostrea gigas*	*Crassostrea*	*Bivalvia*	PCR	ISO/TS 21872-1	Italy	Europe
79	[54]	Tra et al. 2016 (Shrimps, Litopenaeus vannamei, Vietnam)	30	16	Shrimps	*Litopenaeus vannamei*	*Litopenaeus*	*Malacostraca*	PCR	TCBS	Vietnam	Asia
80	[54]	Tra et al. 2016 (Shrimps, P. merguiensis, M. ensis, M. rosenbergii, Vietnam)	5	2	Shrimps	*Penaeus merguiensis, Metapenaeus ensis, Macrobrachium rosenbergii*		*Malacostraca*	PCR	TCBS	Vietnam	Asia
81	[54]	Tra et al. 2016 (Shrimps, Penaeus monodon, Vietnam)	5	2	Shrimps	*Penaeus monodon*	*Penaeus*	*Malacostraca*	PCR	TCBS	Vietnam	Asia
82	[55]	Sperling et al. 2015 (Shrimps, Ecuador)	229	115	Shrimps	NRT	NRT	*Malacostraca*	PCR	ISO/TS 21872-2:2007/TCBS	Ecuador	South America
83	[56]	Kriem et al. 2015 (Shrimps, Parapenaeus longirostris, France)	229	161	Shrimps	*Parapenaeus longirostris*	*Parapenaeus*	*Malacostraca*	PCR	TCBS/CHROMagar™ Vibrio	France	Europe
84	[57]	Xu et al. 2015 (Abalone, China)	50	1	Abalone	NRT	NRT	*Gastropoda*	mPCR-HPLC	TCBS	China	Asia
85	[57]	Xu et al. 2015 (Clams, Corbicula leana, China)	555	1	Clams	*Corbicula leana*	*Corbicula*	*Bivalvia*	mPCR-HPLC	TCBS	China	Asia
86	[57]	Xu et al. 2015 (Clams, Meretrix lusoria, China)	370	0	Clams	*Meretrix lusoria*	*Meretrix*	*Bivalvia*	mPCR-HPLC	TCBS	China	Asia
87	[57]	Xu et al. 2015 (Clams, Ruditapes philippinarum, China)	485	1	Clams	*Ruditapes philippinarum*	*Ruditapes*	*Bivalvia*	mPCR-HPLC	TCBS	China	Asia
88	[57]	Xu et al. 2015 (Clams, Scapharca subcrenata, China)	411	0	Clams	*Scapharca subcrenata*	*Scapharca*	*Bivalvia*	mPCR-HPLC	TCBS	China	Asia
89	[57]	Xu et al. 2015 (Clams, China)	450	5	Clams	NRT	NRT	*Bivalvia*	mPCR-HPLC	TCBS	China	Asia
90	[57]	Xu et al. 2015 (Clams, China)	437	9	Clams	NRT	NRT	*Bivalvia*	mPCR-HPLC	TCBS	China	Asia

91	[57]	Xu et al. 2015 (Crabs, China)	325	1	Crabs	NRT	NRT	*Malacostraca*	mPCR-HPLC	TCBS	China	Asia
92	[57]	Xu et al. 2015 (Crayfish, China)	50	0	Crayfish	NRT	NRT	*Malacostraca*	mPCR-HPLC	TCBS	China	Asia
93	[57]	Xu et al. 2015 (Geoducks, China)	72	0	Geoducks	NRT	NRT	*Bivalvia*	mPCR-HPLC	TCBS	China	Asia
94	[57]	Xu et al. 2015 (Lobsters, China)	50	0	Lobsters	NRT	NRT	*Malacostraca*	mPCR-HPLC	TCBS	China	Asia
95	[57]	Xu et al. 2015 (Mussels, China)	576	2	Mussels	NRT	NRT	*Bivalvia*	mPCR-HPLC	TCBS	China	Asia
96	[57]	Xu et al. 2015 (Oysters, China)	633	10	Oysters	NRT	NRT	*Bivalvia*	mPCR-HPLC	TCBS	China	Asia
97	[57]	Xu et al. 2015 (Prawns, China)	255	0	Prawns	NRT	NRT	*Malacostraca*	mPCR-HPLC	TCBS	China	Asia
98	[57]	Xu et al. 2015 (Scallops, Chlamys farreri, China)	270	0	Scallops	*Chlamys farreri*	*Chlamys*	*Bivalvia*	mPCR-HPLC	TCBS	China	Asia
99	[57]	Xu et al. 2015 (Scallops, China)	592	1	Scallops	NRT	NRT	*Bivalvia*	mPCR-HPLC	TCBS	China	Asia
100	[57]	Xu et al. 2015 (Shrimps, China)	399	2	Shrimps	NRT	NRT	*Malacostraca*	mPCR-HPLC	TCBS	China	Asia
101	[57]	Xu et al. 2015 (Slug, Aplysia, China)	95	0	Slug	*Aplysia*	NRT	*Gastropoda*	mPCR-HPLC	TCBS	China	Asia
102	[57]	Xu et al. 2015 (Snails, Bullacta exarata, China)	210	1	Snails	*Bullacta exarata*	*Bullacta*	*Gastropoda*	mPCR-HPLC	TCBS	China	Asia
103	[57]	Xu et al. 2015 (Squids, China)	95	0	Squids	NRT	NRT	*Cephalopoda*	mPCR-HPLC	TCBS	China	Asia
104	[58]	Khamesipour et al. 2014 (Shrimps, Iran)	36	2	Shrimps	NRT	NRT	*Malacostraca*	PCR	TCBS	Iran	Asia
105	[59]	Govahi et al. 2014 (Shrimps, Litopenaeus vannamei, Iran)	140	20	Shrimps	*Litopenaeus vannamei*	*Litopenaeus*	*Malacostraca*	PCR	Lightner 1996	Iran	Asia
106	[60]	Ramos et al. 2014 (Oysters, Crassostrea gigas, Brazil)	60	3	Oysters	*Crassostrea gigas*	*Crassostrea*	*Bivalvia*	API	TCBS	Brazil	South America
107	[61]	Mannas et al. 2014 (Mussels, Mytilus galloprovincialis, Morocco)	52	47	Mussels	*Mytilus galloprovincialis*	*Mytilus*	*Bivalvia*	API	ISO/TS 16649–3 (2005)/TCBS/CHROMagar™ Vibrio	Morocco	Africa
108	[62]	Raissy et al. 2014 (Crayfish, Astacus leptodactylus, Iran)	97	2	Crayfish	*Astacus leptodactylus*	*Astacus*	*Malacostraca*	PCR	TCBS	Iran	Asia
109	[63]	Elal Mus et al. 2014 (Mussels, Turkey)	12	6	Mussels	NRT	NRT	*Bivalvia*	API	TCBS	Turkey	Europe
110	[63]	Elal Mus et al. 2014 (Shrimps, Turkey)	10	4	Shrimps	NRT	NRT	*Malacostraca*	API	TCBS	Turkey	Europe
111	[64]	Sudha et al. 2014 (Clams, Villorita cyprinoides , India)	19	3	Clams	*Villorita cyprinoides*	*Villorita*	*Bivalvia*	Biochemical test	TCBS	India	Asia
112	[64]	Sudha et al. 2014 (Crabs, Scylla serrata, Portunus pelagicus, Charybdis crussiata, India)	8	0	Crabs	*Scylla serrata, Portunus pelagicus, Charybdis crussiata*		*Malacostraca*	Biochemical test	TCBS	India	Asia
113	[64]	Sudha et al. 2014 (Shrimps, P. indicus, P. monodon, M. dobsonii, M. affinis, P. stylifera, India)	83	10	Shrimps	*Penaeus indicus, Penaeus monodon, M etapenaeus dobsonii, Metapenaeus affinis, Parapenaeopsis stylifera*		*Malacostraca*	Biochemical test	TCBS	India	Asia
114	[65]	Banerjee et al. 2012 (Shrimps, Litopenaeus vannamei, Malaysia)	180	0	Shrimps	*Litopenaeus vannamei*	*Litopenaeus*	*Malacostraca*	API	TCBS	Malaysia	Asia
115	[66]	Cariani et al. 2012 (Clams, Callista chione, Italy)	1	0	Clams	*Callista chione*	*Callista*	*Bivalvia*	PCR	TCBS	Italy	Europe
116	[66]	Cariani et al. 2012 (Clams, Ruditapes philippinarum, Italy)	6	2	Clams	*Ruditapes philippinarum*	*Ruditapes*	*Bivalvia*	PCR	TCBS	Italy	Europe
117	[66]	Cariani et al. 2012 (Mussels, Mytilus galloprovincialis, Italy)	7	4	Mussels	*Mytilus galloprovincialis*	*Mytilus*	*Bivalvia*	PCR	TCBS	Italy	Europe
118	[66]	Cariani et al. 2012 (Oysters, Crassostrea gigas, Italy)	9	1	Oysters	*Crassostrea gigas*	*Crassostrea*	*Bivalvia*	PCR	TCBS	Italy	Europe
119	[67]	Raissy et al. 2012 (Crabs, Iran)	32	1	Crabs	NRT	NRT	*Malacostraca*	PCR	TCBS/mCPC	Iran	Asia
120	[67]	Raissy et al. 2012 (Lobsters, Iran)	100	11	Lobsters	NRT	NRT	*Malacostraca*	PCR	TCBS/mCPC	Iran	Asia
121	[68]	Ramos et al. 2012 (Oysters, Crassostrea gigas, Brazil)	180	15	Oysters	*Crassostrea gigas*	*Crassostrea*	*Bivalvia*	API	TCBS	Brazil	South America

122	[69]	Koralage et al. 2012 (Shrimps, Penaeus monodon, Germany)	170	32	Shrimps	*Penaeus monodon*	*Penaeus*	*Malacostraca*	PCR	TCBS	Germany	Europe
123	[70]	Traore et al. 2012 (Crabs, Callinectes sapidus, Cote d Ivoire)	150	6	Crabs	*Callinectes sapidus*	*Callinectes*	*Malacostraca*	MALDI-TOF MS/PCR	TCBS	Cote d Ivoire	Africa
124	[70]	Traore et al. 2012 (Crabs, Cardisoma, Cote d Ivoire)	20	0	Crabs	*Cardisoma*	*Cardisoma*	*Malacostraca*	MALDI-TOF MS/PCR	TCBS	Cote d Ivoire	Africa
125	[70]	Traore et al. 2012 (Shrimps, Macrobrachium, Cote d Ivoire)	40	0	Shrimps	*Macrobrachium*	*Macrobrachium*	*Malacostraca*	MALDI-TOF MS/PCR	TCBS	Cote d Ivoire	Africa
126	[70]	Traore et al. 2012 (Shrimps, Penaeus, Cote d Ivoire)	112	4	Shrimps	*Penaeus*	*Penaeus*	*Malacostraca*	MALDI-TOF MS/PCR	TCBS	Cote d Ivoire	Africa
127	[71]	Zarei et al. 2012 (Shrimps, Iran)	300	53	Shrimps	NRT	NRT	*Malacostraca*	PCR	TCBS	Iran	Asia
128	[72]	Raissy et al. 2011 (Lobsters, Panulirus homarus, Iran)	60	4	Lobsters	*Panulirus homarus*	*Panulirus*	*Malacostraca*	PCR	TCBS	Iran	Asia
129	[73]	Scharer et al. 2011 (Bivalves, Switzerland)	34	26	Bivalves	NRT	NRT	*Bivalvia*	PCR	TCBS	Switzerland	Europe
130	[73]	Scharer et al. 2011 (Squids, Switzerland)	2	2	Squids	NRT	NRT	*Cephalopoda*	PCR	TCBS	Switzerland	Europe
131	[77]	Eja et al. 2008 (Clams, Egeria radiata, Nigeria)	90	4	Clams	*Egeria radiata*	*Egeria*	*Bivalvia*	Biochemical test	TCBS	Nigeria	Africa
132	[77]	Eja et al. 2008 (Perwinkles, Tympanotonus fuscatus, Nigeria)	98	6	Perwinkles	*Tympanotonus fuscatus*	*Tympanotonus*	*Gastropoda*	Biochemical test	TCBS	Nigeria	Africa
133	[77]	Eja et al. 2008 (Shrimps, Macrobrachium vollenhovenii, Nigeria)	120	12	Shrimps	*Macrobrachium vollenhovenii*	*Macrobrachium*	*Malacostraca*	Biochemical test	TCBS	Nigeria	Africa
134	[75]	Zhou et al. 2007 (Oysters, China)	8	8	Oysters	NRT	NRT	*Bivalvia*	qPCR	TCBS	China	Asia
135	[75]	Zhou et al. 2007 (Shrimps, China)	8	8	Shrimps	NRT	NRT	*Malacostraca*	qPCR	TCBS	China	Asia
136	[76]	Cohen et al. 2007 (Mussels, Morocco)	25	2	Mussels	NRT	NRT	*Bivalvia*	PCR	TCBS	Morocco	Africa
137	[76]	Cohen et al. 2007 (Oysters, Morocco)	25	14	Oysters	NRT	NRT	*Bivalvia*	PCR	TCBS	Morocco	Africa
138	[76]	Cohen et al. 2007 (Prawns, Morocco)	35	2	Prawns	NRT	NRT	*Malacostraca*	PCR	TCBS	Morocco	Africa
139	[76]	Cohen et al. 2007 (Squids, Morocco)	35	0	Squids	NRT	NRT	*Cephalopoda*	PCR	TCBS	Morocco	Africa
140	[77]	Vigano et al. 2007 (octopus, squid, clam,mussells, Tanzania)	85	13	octopus, squid, clam,mussells	NRT	NRT	*NRT*	API	TCBS	Tanzania	Africa
141	[77]	Vigano et al. 2007 (Prawns, Tanzania)	8	2	Prawns	NRT	NRT	*Malacostraca*	API	TCBS	Tanzania	Africa
142	[78]	Pereira et al. 2007 (Mussels, Perna perna, Brazil)	86	31	Mussels	*Perna perna*	*Perna*	*Bivalvia*	Biochemical test	TCBS	Brazil	South America
143	[79]	Colakoglu et al. 2006 (Mussels, Mytilus galloprovincialis, Donax trunculus, Turkey)	100	22	Mussels	*Mytilus galloprovincialis, Donax trunculus*		*Bivalvia*	API	TCBS	Turkey	Europe
144	[79]	Colakoglu et al. 2006 (Prawns, Parapenaeus longirostris, Turkey)	27	12	Prawns	*Parapenaeus longirostris*	*Parapenaeus*	*Malacostraca*	API	TCBS	Turkey	Europe
145	[80]	Hosseini et al. 2004 (Shrimps, Iran)	770	4	Shrimps	NRT	NRT	*Malacostraca*	Biochemical test	TCBS	Iran	Asia
146	[81]	Elhadi et al. 2004 (Cockles, Anadara granosa, Japan)	50	7	Cockles	*Anadara granosa*	*Anadara*	*Bivalvia*	Biochemical test	TCBS	Japan	Asia
147	[81]	Elhadi et al. 2004 (Cockles, Lithophaga malaccana, Japan)	50	7	Cockles	*Lithophaga malaccana*	*Lithophaga*	*Bivalvia*	Biochemical test	TCBS	Japan	Asia
148	[81]	Elhadi et al. 2004 (Crabs, Japan)	50	9	Crabs	NRT	NRT	*Malacostraca*	Biochemical test	TCBS	Japan	Asia
149	[81]	Elhadi et al. 2004 (Mussels, Japan)	50	2	Mussels	NRT	NRT	*Bivalvia*	Biochemical test	TCBS	Japan	Asia
150	[81]	Elhadi et al. 2004 (Shrimps, Japan)	433	43	Shrimps	NRT	NRT	*Malacostraca*	Biochemical test	TCBS	Japan	Asia
151	[81]	Elhadi et al. 2004 (Squids, Loligo spp., Japan)	75	9	Squids	*Loligo spp.*	*Loligo*	*Cephalopoda*	Biochemical test	TCBS	Japan	Asia
152	[81]	Elhadi et al. 2004 (Squids, Spia sp., Japan)	60	5	Squids	*Spia sp.*	*Spia*	*Cephalopoda*	Biochemical test	TCBS	Japan	Asia

153	[82]	Yalcinkaya et al. 2003 (Crabs, Callinectes sapidus, Turkey)	83	25	Crabs	*Callinectes sapidus*	Callinectes	Malacostraca	Biochemical test	TCBS		Turkey	Europe
154	[83]	Ndip et al. 2002 (Shrimps, Macrobranchium spp, Cameroon)	236	34	Shrimps	*Macrobranchium spp*	Macrobranchium	Malacostraca	API	TCBS		Cameroon	Africa
155	[84]	Jaksic et al. 2002 (Bivalves, Croatia)	29	2	Bivalves	NRT	NRT	Bivalvia	API	TCBS		Croatia	Europe
156	[84]	Jaksic et al. 2002 (Shrimps, Croatia)	27	1	Shrimps	NRT	NRT	Malacostraca	API	TCBS		Croatia	Europe
157	[85]	Baffone et al. 2000 (Clams, Venus gallina, Italy)	49	4	Clams	*Venus gallina*	Venus	Bivalvia	Biochemical test	TCBS		Italy	Europe
158	[85]	Baffone et al. 2000 (Mussels, Mytilus galloprovincialis, Italy)	37	6	Mussels	*Mytilus galloprovincialis*	Mytilus	Bivalvia	Biochemical test	TCBS		Italy	Europe
159	[86]	Ripabelli et al. 1999 (Mussels, Mytilus galloprovincialis, Italy)	62	20	Mussels	*Mytilus galloprovincialis*	Mytilus	Bivalvia	Biochemical test	TCBS		Italy	Europe
160	[87]	Hariharan et al. 1995 (Mussels, Canada)	85	6	Mussels	NRT	NRT	Bivalvia	Biochemical test	TCBS		Canada	North America
161	[87]	Hariharan et al. 1995 (Oysters, Canada)	46	0	Oysters	NRT	NRT	Bivalvia	Biochemical test	TCBS		Canada	North America
162	[88]	Sunen et al. 1995 (Clams, Spain)	21	9	Clams	NRT	NRT	Bivalvia	Biochemical test	TCBS		Spain	Europe
163	[88]	Sunen et al. 1995 (Mussels, Spain)	22	13	Mussels	NRT	NRT	Bivalvia	Biochemical test	TCBS		Spain	Europe
164	[89]	Wong et al. 1995 (Shrimps, China)	74	23	Shrimps	NRT	NRT	Malacostraca	Biochemical test	TCBS		China	Asia
165	[90]	Matte et al. 1994 (Oysters, Crassostrea gigas, Brazil)	26	21	Oysters	*Crassostrea gigas*	Crassostrea	Bivalvia	Biochemical test	TCBS		Brazil	South America
166	[90]	Matte et al. 1994 (Mussels, Perna perna, Brazil)	36	35	Mussels	*Perna perna*	Perna	Bivalvia	Biochemical test	TCBS		Brazil	South America
167	[91]	Wong et al. 1992 (Clams, Corbicula fluminea, China)	14	8	Clams	*Corbicula fluminea*	Corbicula	Bivalvia	Biochemical test	TCBS		China	Asia
168	[91]	Wong et al. 1992 (Clams, Meretrix lusoria, China)	20	12	Clams	*Meretrix lusoria*	Meretrix	Bivalvia	Biochemical test	TCBS		China	Asia
169	[91]	Wong et al. 1992 (Crabs, Portunus spp., China)	12	8	Crabs	*Portunus spp.*	Portunus	Malacostraca	Biochemical test	TCBS		China	Asia
170	[91]	Wong et al. 1992 (Oysters, Crassostrea gigas, China)	16	11	Oysters	*Crassostrea gigas*	Crassostrea	Bivalvia	Biochemical test	TCBS		China	Asia
171	[91]	Wong et al. 1992 (Shrimps, Penaeus monodon, Penaeus spp, China)	47	33	Shrimps	*Penaeus monodon, Penaeus spp*	Penaeus	Malacostraca	Biochemical test	TCBS		China	Asia
172	[92]	Colburn et al. 1989 (Oysters, Crassostrea gigas, USA)	27	0	Oysters	*Crassostrea gigas*	Crassostrea	Bivalvia	API	TCBS		USA	North America
173	[93]	Chan et al. 1989 (Clams, Scapharca cornea, China)	50	8	Clams	*Scapharca cornea*	Scapharca	Bivalvia	API	TCBS		China	Asia
174	[93]	Chan et al. 1989 (Crabs, Scylla serrata, China)	35	4	Crabs	*Scylla serrata*	Scylla	Malacostraca	API	TCBS		China	Asia
175	[93]	Chan et al. 1989 (Mussels, Perna viridis, China)	50	10	Mussels	*Perna viridis*	Perna	Bivalvia	API	TCBS		China	Asia
176	[93]	Chan et al. 1989 (Oysters, Crassostrea gigas, China)	50	18	Oysters	*Crassostrea gigas*	Crassostrea	Bivalvia	API	TCBS		China	Asia
177	[93]	Chan et al. 1989 (Prawns, Penaeus orientalis, China)	50	4	Prawns	*Fenneropenaeus chinensis*	*Fenneropenaeus*	Malacostraca	API	TCBS		China	Asia
178	[94]	Utsalo et al. 1988 (Clams, Mercenaria spp, Nigeria)	110	64	Clams	*Mercenaria* spp	Mercenaria	Bivalvia	Biochemical test	TCBS		Nigeria	Africa
179	[95]	Kampelmacher et al. 1972 (Mussels, Netherlands)	144	5	Mussels	NRT	NRT	Bivalvia	Biochemical test	TCBS		Netherlands	Europe
180	[95]	Kampelmacher et al. 1972 (Oysters, Netherlands)	44	3	Oysters	NRT	NRT	Bivalvia	Biochemical test	TCBS		Netherlands	Europe

Penaeus vannamei = Litopenaeus vannamei   and Penaeus orientalis = Fenneropenaeus chinensis species-specific prevalence analysi
